# T-cell metagene predicts a favorable prognosis in estrogen receptor-negative and HER2-positive breast cancers

**DOI:** 10.1186/bcr2234

**Published:** 2009-03-09

**Authors:** Achim Rody, Uwe Holtrich, Laos Pusztai, Cornelia Liedtke, Regine Gaetje, Eugen Ruckhaeberle, Christine Solbach, Lars Hanker, Andre Ahr, Dirk Metzler, Knut Engels, Thomas Karn, Manfred Kaufmann

**Affiliations:** 1 Department of Obstetrics and Gynecology, J.W. Goethe-University, Theodor-Stern-Kai 7, Frankfurt 60590, Germany; 2 Department of Breast Medical Oncology, The University of Texas M.D. Anderson Cancer Center, PO Box 301439, Houston, TX 77230-1439, USA; 3 Department of Obstetrics and Gynecology, University of Muenster, Albert-Schweitzer-Straße 33, Muenster 48149, Germany; 4 LMU BioCenter, Ludwig Maximilians University Munich, Grosshaderner Straße 2, Planegg-Martinsried 82152, Germany; 5 Department of Pathology, J.W. Goethe-University, Theodor-Stern-Kai 7, Frankfurt 60590, Germany

## Abstract

**Introduction:**

Lymphocyte infiltration (LI) is often seen in breast cancer but its importance remains controversial. A positive correlation of human epidermal growth factor receptor 2 (HER2) amplification and LI has been described, which was associated with a more favorable outcome. However, specific lymphocytes might also promote tumor progression by shifting the cytokine milieu in the tumor.

**Methods:**

Affymetrix HG-U133A microarray data of 1,781 primary breast cancer samples from 12 datasets were included. The correlation of immune system-related metagenes with different immune cells, clinical parameters, and survival was analyzed.

**Results:**

A large cluster of nearly 600 genes with functions in immune cells was consistently obtained in all datasets. Seven robust metagenes from this cluster can act as surrogate markers for the amount of different immune cell types in the breast cancer sample. An IgG metagene as a marker for B cells had no significant prognostic value. In contrast, a strong positive prognostic value for the T-cell surrogate marker (lymphocyte-specific kinase (LCK) metagene) was observed among all estrogen receptor (ER)-negative tumors and those ER-positive tumors with a HER2 overexpression. Moreover ER-negative tumors with high expression of both IgG and LCK metagenes seem to respond better to neoadjuvant chemotherapy.

**Conclusions:**

Precise definitions of the specific subtypes of immune cells in the tumor can be accomplished from microarray data. These surrogate markers define subgroups of tumors with different prognosis. Importantly, all known prognostic gene signatures uniformly assign poor prognosis to all ER-negative tumors. In contrast, the LCK metagene actually separates the ER-negative group into better or worse prognosis.

## Introduction

There is growing evidence that interaction of stromal and immune cells with normal or malignant epithelial cells is pivotal for the development and progression of cancer. Several reports indicate that tumor-infiltrating leucocytes may represent an essential pathophysiological factor in the development and progression of breast cancer [1-3]. Their prognostic impact, however, remains unclear. Lymphocyte infiltration (LI) is often seen in breast cancer and has been suggested as a marker of host antitumor immune response, but its importance in terms of pathophysiology and prognosis or treatment prediction remains controversial. The presence of B cells is already seen with premalignant breast tumors [4], while T-cell infiltration is associated only with high-grade ductal carcinoma *in situ *and invasive carcinoma [5] and has been reported to range from 1% to 45% of the total cellular mass [6].

In rapidly proliferating tumors LI has been shown to be a good prognostic indicator, correlating with lymph-node negativity, smaller tumor size, and lower grade [7]. Similarly, Ménard and colleagues have shown that lymphocyte infiltration of breast cancer had a strong positive prognostic value in patients younger than 40 years; no association was seen among patients 40 years or older, however, suggesting a correlation with estrogen receptor (ER) status or specific breast cancer subtypes [8]. A positive correlation of human epidermal growth factor receptor 2 (HER2) amplification/overexpression, LI and expression of lymphocyte-associated genes has been described that was associated with a more favorable outcome [9]. Only a small fraction of tumor-associated lymphocytes display activation markers, however, and there is no definitive proof of cytotoxic activity of these cells against the tumor *in vivo *[10]. In this context the expression of specific oncoproteins such as HER2 or p53 is supposed to be immunogenic [11].

The search for prognostic or predictive signatures using microarray analysis in bulk breast cancer specimens reveals several genes that are associated with immune cells; for example, interferon-regulated genes [12,13], B-lymphocyte marker [12], as well as T-lymphocyte-associated genes [13]. In this context, whether these observations are due to an imbalance of host-associated markers and tumor tissue or due to a real biological phenomenon remains unclear. Data from gene expression profiling of breast cancer cell lines showed that a considerable number of immune-response-related genes exhibit significant variable expression across the basal cell subtype [14,15], suggesting that immune response genes might play a crucial role even in the absence of host cells.

Most recently, Finak and colleagues identified a good-outcome cluster from gene expression profiles of tumor stroma that was isolated by laser-captured microdissection. This cluster contained 22 different genes 'enriched for elements of the T helper type 1 (TH1) immune response' of which the authors verified selected markers by immunohistochemistry [16].

Overall, the impact of monocytes, B lymphocytes and T lymphocytes on prognosis is still a matter of debate. The purpose of our study was therefore to accurately identify different clusters of immune-cell-associated genes in bulk breast cancer samples by a large-scale analysis of microarray datasets, and to precisely analyze the correlation between the resulting metagenes and specific breast cancer subtypes. Finally, we evaluated the prognostic impact of these metagenes in defined breast cancer subgroups.

## Materials and methods

### Microarray data

A database of 1,781 primary invasive breast cancers including all samples from 12 Affymetrix HG-U133 microarray datasets was established: Frankfurt [17,18] (Additional data file 1), Uppsala [19], Oxford – Untreated [20], Stockholm [21], New York [22], London [23], Rotterdam [24,25], Oxford – Tamoxifen and Villejuif [26], Expression Project for Oncology [27], Frankfurt-2 [28] (Additional data file 1), and MDA133 [29]. Characteristics of the individual datasets are presented in Additional data file 1. Follow-up information was available for 1,263 patients. The median follow-up time was 79 months. Seventy-two percent of all samples and 74% of those samples with follow-up were ER-positive.

Only Affymetrix HG-U133A microarrays were included for full comparability of all the probes on the arrays. Data were downloaded from the Gene Expression Omnibus website [30]. Affymetrix expression data for different immunological cell types and tissues were obtained from Su and colleagues [GEO:GSE1133] [31]. Affymetrix expression data were analyzed using the MAS5.0 algorithm [32] of the *affy *package [33] from the Bioconductor software project [34]. Expression data were log_2_-transformed and were normalized across each individual array by a scaling factor *S *so that the magnitude (sum of the squares of the values) equals one.

### Metagenes for feature reduction

A high feature-to-sample ratio is one of the most important problems in microarray research leading to an inflation of α values [35]. Unsupervised clustering was applied for feature reduction based on the assumption that the expression of a large number of genes is highly interdependent. This can be attributed to the expression of sets of genes in different cell types in the sample and to differentiation programs/pathways associated with specific expression profiles. Genes that did not show a correlation with other genes above a certain threshold (0.7) were suspected to represent noise, and were discarded from further analysis. To identify metagenes for the principal vectors, we selected those clusters that contained at least 10 elements and a minimal average correlation of 0.7 – resulting in 199 total ProbeSets. Metagene expression values were determined by calculating the mean of the normalized expression values of all ProbeSets in the respective cluster.

### Assessment of ER, HER2, proliferative status and tumors with stem-cell-like characteristics of the samples

To allow comparative analysis between different datasets and since standard pathology for ER and HER2 was not available for all samples, the receptor status was determined based on Affymetrix expression data as previously described [36]. A stem-cell-like (SCL) metagene was used as described previously [37,38]. This metagene was derived from 159 highly correlated Affymetrix ProbeSets and contains 35 out of 37 (95%) previously reported markers of SCL breast cancers, undifferentiated breast cancers and basal-like breast cancers [39-43].

### Immunohistochemistry

To validate the presence of lymphocytes in those samples that show a high expression of the respective metagenes, we performed immunohistochemistry using specific antibodies. CD3 (clone F7.2.38; Dianova, Hamburg, Germany) and CD20 (clone B-Ly1; Dianova) were used as markers for T lymophocytes and B lymphocytes, respectively.

Tissue samples of primary invasive breast cancer cases from the University of Frankfurt were obtained with informed consent and approval of the institutional review board of the University of Frankfurt. Briefly, paraffin sections (2 μm) were mounted on Superfrost Plus slides, dewaxed in xylene and rehydrated through graduated ethanol to water. Antigens were retrieved by microwaving sections in 10 mM citrate buffer (pH 6.0) for 20 minutes at 800 W. Blocking was performed using antibody dilution buffer (DCS Diagnostics, Hamburg, Germany) at room temperature for 15 minutes. Antibodies were subsequently diluted 1:100 individually in this buffer. Sections were incubated with antibodies for 1 hour at room temperature.

For negative controls, the primary antibodies were replaced with PBS. For secondary antibody incubations and detection, the Dako REAL Detection System Alkaline Phosphatase/RED (Dako, Glostrup, Denmark) was used following the protocol of the supplier and sections were counterstained with Mayer's hematoxylin. Samples from the Frankfurt dataset were ranked according to visual inspection of the amount of stained lymphocytes with the respective antibody in a blinded analysis. The rank order was subsequently compared with that based on the metagene expression using Spearman rank correlation.

### Statistical analyses

All reported *P *values are two sided and *P *< 0.05 was considered to indicate a significant result. Subjects with missing values were excluded from the analyses. Fisher's exact test was applied for associations between categorical parameters. Spearman rank correlation was used to compare metagene expression and results from immunohistochemistry. The Kruskal–Wallis H test was used to analyze the relationship of the expression of immune metagenes and pathological lymphocyte infiltration scores from the independent validation dataset from London.

Survival intervals were measured from the time of surgery to the time of death from disease or of the first clinical or radiographic evidence of disease recurrence. Data for women in whom the envisaged end point was not reached were censored as of the last follow-up date or at 120 months. We constructed Kaplan–Meier curves and used the log-rank test to determine the univariate significance of the variables. Hazard ratios were determined by Cox regression.

To examine simultaneously the effects of multiple standard parameters and lymphocyte-specific kinase (LCK) metagene expression on survival, a Cox proportional-hazards regression model was applied among ER-negative samples. The effect of each variable was assessed with the use of the Wald test and described by the hazard ratio with a 95% confidence interval. The model included binary variables for lymph node status (lymph node-negative or N1), histological grading (G1 or G2 vs. G3), age (≤ 50 years vs. > 50 years), tumor size (≤ 2 cm vs. > 2 cm), and HER2 status (by microarray [36]). All analyses were performed using SPSS 15.0 (SPSS Inc., Chicago, IL, USA) and R 2.6.2 software [44].

## Results

Unsupervised hierarchical clustering of genes in individual datasets as well as combined datasets revealed a large cluster of genes with functions in immune cells. This cluster of approximately 600 Affymetrix ProbeSets was consistently obtained in all datasets with overall correlations of 0.2 to 0.3. We hypothesized that the observed coordinated expression of subsets of these genes might represent surrogate markers for the amounts of different types of immune cells in the analyzed samples. In addition, coordinated expression might result from the induction of signaling pathways and specific differentiation programs in the tumor cells themselves and/or accompanying stromal tissue.

**Figure 1:**
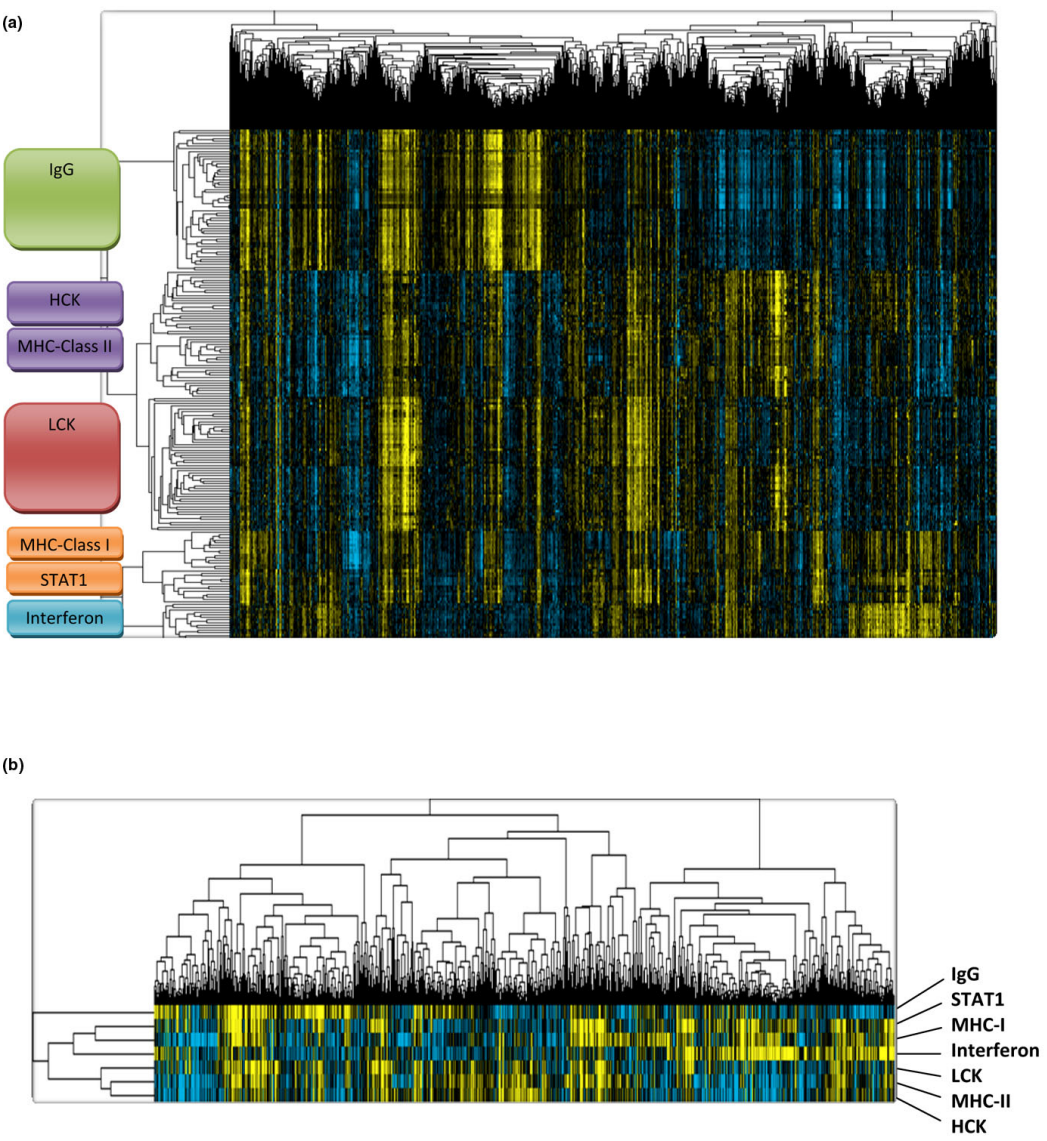
Identification of immune-system-related metagenes. **(a) **To identify metagenes for the principal expression vectors we selected those gene clusters that encompassed at least 10 elements and displayed a minimal average correlation of 0.7 from the larger data matrix of 569 ProbeSets (see Additional data file 3). Expression of these selected 199 ProbeSets among the 1,230 breast cancer samples is shown. HCK, hemopoietic cell kinase; LCK, lymphocyte-specific kinase; MHC, major histocompatibility complex; STAT1, signal transducer and activator of transcription 1. **(b) **Seven metagenes were derived as mean values of all 199 ProbeSets from the seven clusters.

The expression of 569 Affymetrix ProbeSets from the immune-related cluster was analyzed in a combined cohort of 1,230 samples to tease out relationships of these genes (see Additional data files 2, 3 and 4). To identify metagenes for the principal expression vectors we selected those clusters that contained at least 10 elements and a minimal average correlation of 0.7, resulting in 199 total ProbeSets as shown in Figure 1a. Seven metagenes were derived as mean values of all ProbeSets in the respective clusters (Figure 1b). The functional annotation of the immune-system-related metagene clusters is presented in Table 1 (a detailed list of all 199 ProbeSets is given in Additional data file 5).

**Table 1:** Functional annotation of the immune-system-related metagene clusters

Metagene	Incorporated genes^a^
IgG	Most of the genes in this cluster represent genes of immunoglobulins of the immunoglobulin gamma type mainly associated with B lymphocytes
HCK	This cluster encompasses genes specific for macrophages and cells of the monocyte/myeloid lineage such as hemopoietic cell kinase, CD163, Chemokine (C-C motif) receptor 1 (CCR1), complement receptors, and B7-2
MHC-II	This cluster contains the HLA-DP, HLA-DQ, HLA-DR genes of the major histocompatability class II complex expressed on the surface of professional antigen-presenting cells for their interaction with T cells
LCK	Genes in this cluster contain T-cell-specific markers such as CD3, T-cell receptor α, T-cell receptor β, lymphocyte-specific kinase, IL-7 receptor
MHC-I	This cluster contains HLA-A, HLA-B, HLA-C, HLA-F and HLA-G genes of the major histocompatability class I complex common to all cell types for the presentation of intracellular antigens
STAT1	The genes in this cluster are associated with interferon signal transduction (like signal transducer and activator of transcription 1 and interferon regulatory factor 1 (IRF1)) and are also interferon inducible
Interferon	All genes in this cluster represent genes known to be interferon inducible and that are associated with the interferon response of cells

^a^See Additional data file 5 for a detailed complete list of all genes.

### Expression of the metagene clusters in different immunological cell types

**Figure 2:**
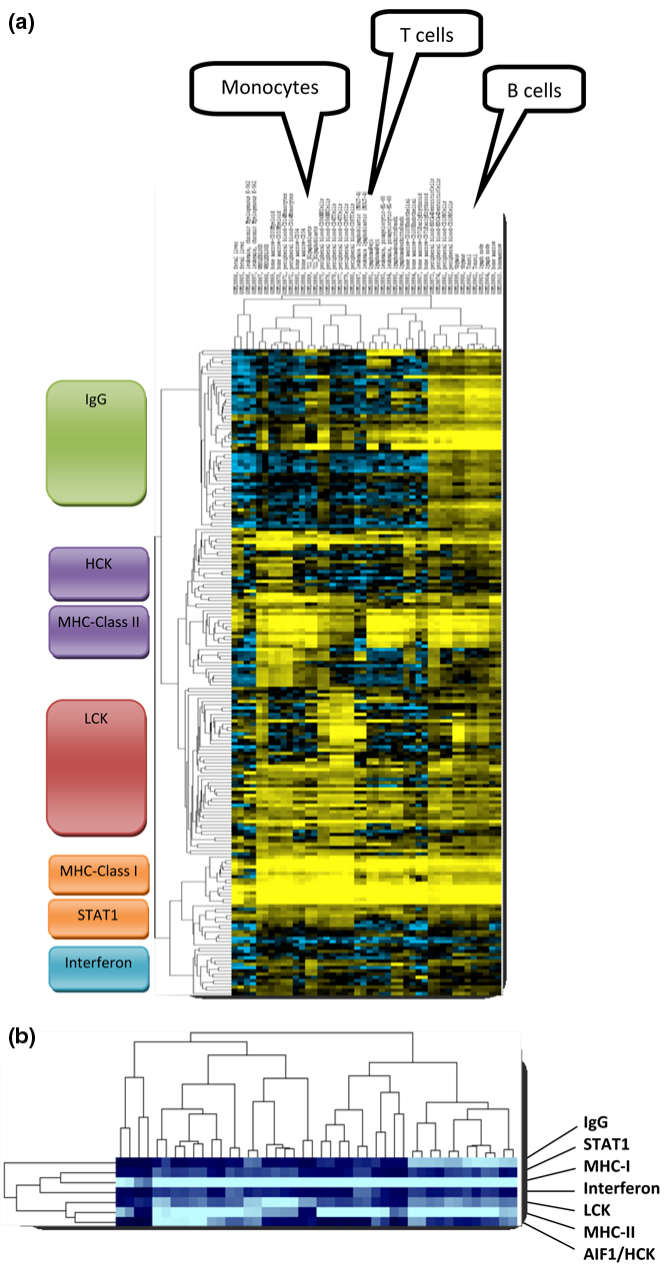
Expression of the metagene clusters in immunological cell types. **(a) **The 199 ProbeSets from Figure 1a were used to cluster 44 samples of isolated cells and tissues with immune-system-related functions that were profiled on Affymetrix U133A arrays by Su and colleagues [GEO:GSE1133] [31]. In each case, two samples for the following cell/tissue types are presented from left to right: fetal liver (1,2), K-562 (3,4), whole blood (5,6), CD33 myeloid (7,8), CD14 monocytes (9,10), CD34 (11,12), B lymphoblasts (13,14), CD56 natural killer cells (15,16), CD4 T cells (17,18), CD8 T cells (19,20), MOLT-4 (21,22), Raji (23,24), HL-60 (25,26), Daudi (27,28), CD105 (29,30), CD71 (31,32), BDCA4 dendritic cells (33,34), CD19 B cells (35,36), thymus (37,38), tonsil (39,40), lymph node (41,42), bone marrow (43,44). Details about the respective samples are given in Additional data file 10. HCK, hemopoietic cell kinase; LCK, lymphocyte-specific kinase; MHC, major histocompatibility complex; STAT1, signal transducer and activator of transcription 1. **(b) **Representation of the seven metagenes that were derived from the 199 ProbeSets as in Figure 1b.

To check the biological plausibility of the identified metagenes as markers for cell types and/or an immunological state, we analyzed their expression in different types of immune system tissues and cell types (Figure 2). As expected, the IgG metagene cluster seemed to be specific for B cells and those tissues containing high amounts of these cells (tonsils, lymph nodes, bone marrow). The hemopoietic cell kinase metagene cluster displayed highest expression in peripheral blood CD14 monocytes and bone-marrow-derived CD33 myeloid cells, in line with the well-known function of the *hck *gene in this lineage. In contrast it is important to note that T cells of both the CD4 and CD8 types are devoid of the expression of this metagene (while some lower levels of expression are detected in the B-cell lineage). Inversely to hemopoietic cell kinase, the LCK metagene is expressed only in T cells but no expression is observed in monocytes and the myeloid lineage. The MHC-II metagene is only expressed by antigen-presenting cells but not in T cells, while high expression of the MHC-I metagene is observed in all cell types as expected. The differences in the interferon and signal transducer and activator of transcription 1 (STAT1) metagenes are smaller than those observed between different tumor samples, which might suggest considerable expression of those interferon-induced genes by the carcinoma and/or stromal cells of the tumor.

### Relationship of expression of immune-system-related metagenes with ER status, HER2 status and presence of stem-cell-like markers and lymphocyte infiltration

To analyze the relationship of the immune system metagenes and standard parameters, unsupervised clustering analysis of all 1,781 samples using the immune-related metagenes, ER, HER2 as well as a metagene of SCL markers was performed (Additional data file 6, Supplemental figure S2a). The results suggested that considerable amounts of immune cells are present among all different subtypes of tumors. Similar results were obtained in the analysis of scatter plots comparing five metagenes representing the major clusters (LCK, IgG, MHC-II, interferon, STAT1) and ER and HER2 status (Additional data file 6, Supplemental figure S2b). The scatter of LCK and IgG (as well as MHC-II) metagenes showed a correlation (*R*^2 ^= 0.52 and *R*^2 ^= 0.62, respectively) that was not observed between the interferon and IgG metagenes (*R*^2 ^= 0.07). This could suggest a parallel infiltration by both T cells and B cells into those tumors that are characterized by high expression of both metagenes. On the other hand, the interferon and STAT1 metagenes are also correlated (*R*^2 ^= 0.52), which might represent an interferon response of tumor cells or other cell types in the respective samples. In general, no clear relationship with ER and HER2 status was seen in these scatter plots. ER-negative tumors, however, display a somewhat higher expression of the IgG and STAT1 metagenes.

**Figure 3:**
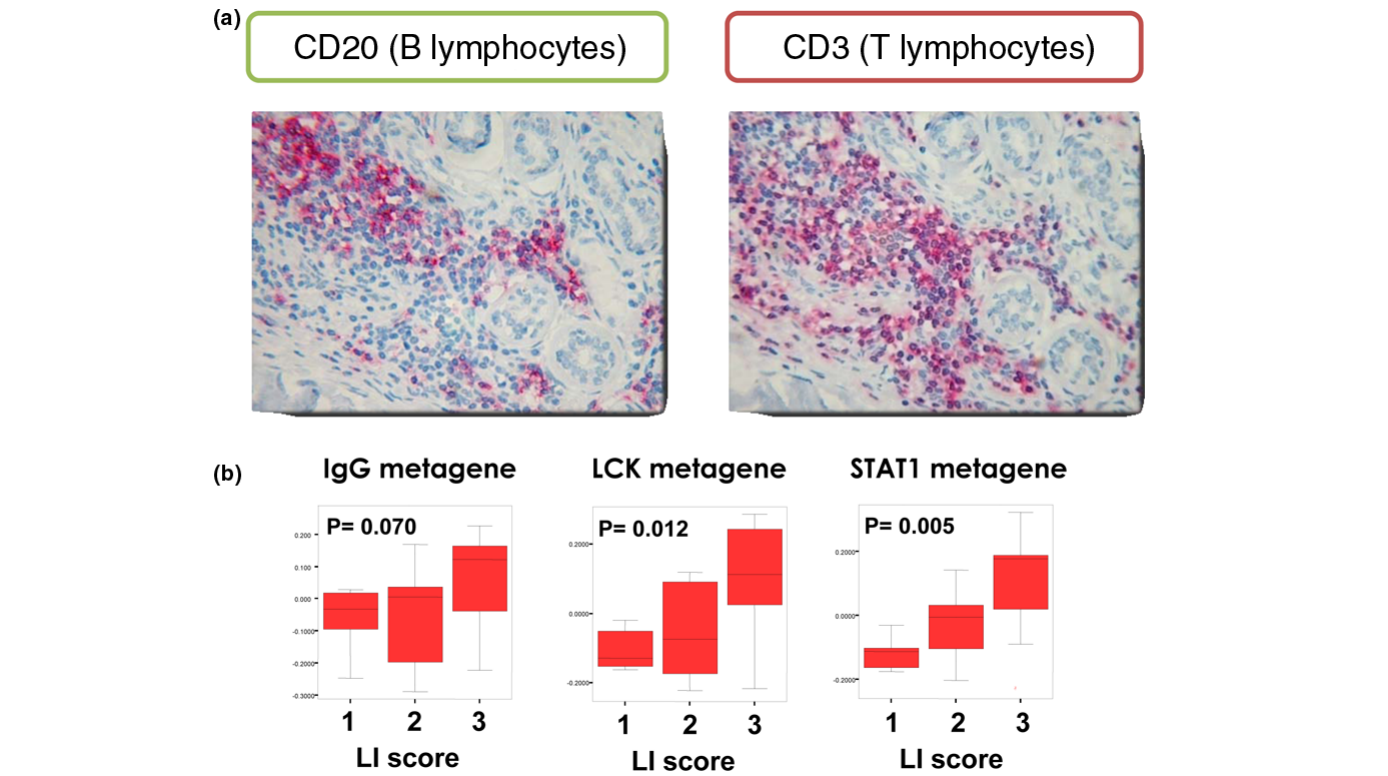
Verification of microarray results by histological examination. **(a) **Example of the verification of lymphocytic infiltration by immunohistochemistry (Frankfurt dataset). Consecutive sections of a tumor sample with high expression of both IgG and lymphocyte-specific kinase (LCK) metagenes stained with antibodies against either CD20 or CD3 to detect B lymphocytes and T lymphocytes, respectively. **(b) **Validation of the correlation of immune-system-related metagenes and lymphocytic infiltration in independent data. Expression of different metagenes compared with pathological information on lymphocytic infiltration (LI score) from the London dataset (Desmedt and colleagues [26], n = 35). *P *values determined using the Kruskal–Wallis H test. STAT1, signal transducer and activator of transcription 1.

To verify the actual presence of lymphocytes in those samples that show a high expression of the respective metagenes we performed immunohistochemistry using specific antibodies. CD3 and CD20 were used as markers for T lymphocytes and B lymphocytes, respectively. Using 10 samples from our own dataset we observed Spearman rank correlations of 0.79 (*P *= 0.006) between CD3 and the LCK metagene and of 0.64 (*P *= 0.048) between CD20 and the IgG metagene, respectively. Figure 3a presents a sample with high expression of both the LCK and IgG metagenes that was characterized by high numbers of T cells and B cells in the sample. For an independent validation of the results we used a dataset from London (Desmedt and colleagues' dataset GUYU [26]). For 35 samples of this dataset, pathological information on lymphocytic infiltration was available. As shown in Figure 3b, a higher expression of all metagenes was detected in those samples with higher scoring for lymphocytic infiltration.

### Prognostic value of immune-system-related metagenes in subgroups of breast cancer patients

There are somewhat differing data in the literature on the frequency of lymphocyte infiltration as indicated by pathological analysis. While earlier studies reported frequencies of 20% (n = 382) [45] up to 45% (n = 78) [46], more recent studies have reported proportions of 16% to 17% (n = 1,919) [8] and 24% (n = 675) [47]. Specific detection of B-lymphocyte infiltration has been reported for 20% of invasive breast carcinomas [4,48]. Bearing these data in mind we used the upper quartile (25%) of the samples with highest expression of the respective metagenes to define a cutoff point for sample stratification. In addition, verification of the robustness by applying simple median splits of the cohorts led to similar results (data not shown).

**Figure 4:**
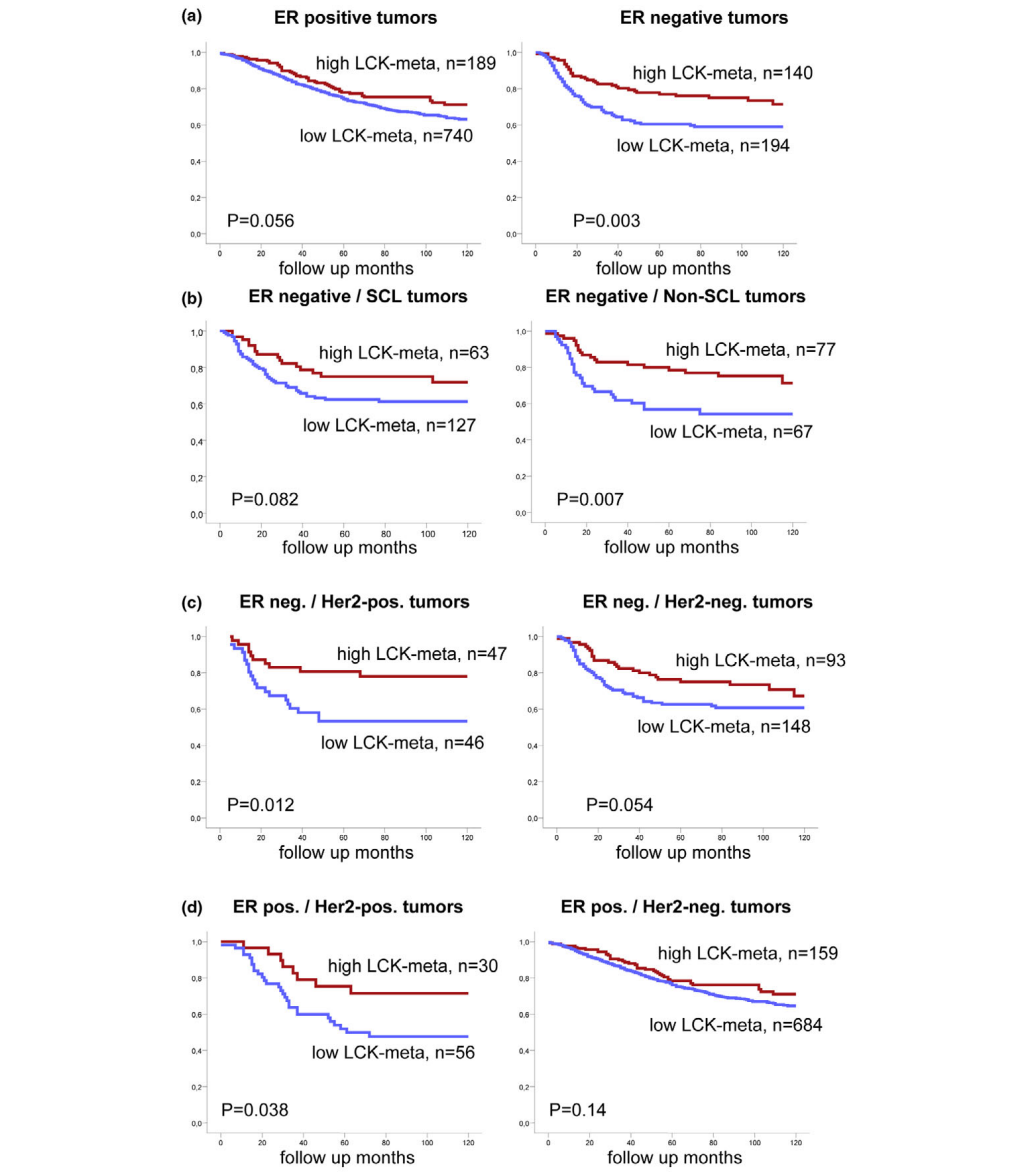
Prognostic value of the lymphocyte-specific kinase metagene in subgroups of breast cancer patients. Samples of the combined dataset were stratified according to the highest quartile of expression of the lymphocyte-specific kinase (LCK) metagene. Kaplan–Meier analyses of disease-free survival were performed in different tumor subgroups according to estrogen receptor (ER), human epidermal growth factor receptor 2 (HER2), and stem-cell like (SCL) status. **(a) **The LCK metagene had a highly significant prognostic value among ER-negative samples. This high prognostic value was observed in all ER-negative samples independently of **(b) **their expression of SCL markers or **(c) **their HER2 status. **(d) **In addition, a high prognostic value of LCK metagene expression was also found in ER-positive HER2-positive samples.

Follow-up information was available for 1,263 out of the 1,781 samples; 929 of these samples were ER-positive and 334 samples were ER-negative. We used multivariate Cox regression among these 1,263 patients to analyze whether the seven metagenes provide prognostic information independent from one another (Additional data file 7). Only for the LCK metagene was a significant result obtained in this analysis (hazards ratio = 0.6, 95% confidence interval = 0.39 to 0.89; *P *= 0.013), while merely a trend was observed for MHC-I (*P *= 0.11) and STAT1 (*P *= 0.13) metagenes. To identify those samples where expression of the LCK metagene has the largest impact on prognosis we performed Kaplan–Meier analyses of disease-free survival in different tumor subgroups stratified by ER, HER2, and SCL status. As shown in Figure 4a, the LCK metagene had the highest prognostic value among the 334 ER-negative samples with a univariate hazard ratio of 1.81 (95% confidence interval = 1.22 to 2.71, *P *= 0.003). This high prognostic value was observed in all ER-negative samples independently of their expression of SCL markers (Figure 4b) or their HER2 status (Figure 4c). In addition a high prognostic value of LCK metagene expression was also found among those 86 ER-positive samples that were also HER2-positive (ER^+^/HER2^+^, *P *= 0.038) (Figure 4d).

To demonstrate that the LCK metagene was an independent prognostic factor and not a surrogate marker for other factors, we performed a multivariate Cox regression analysis including all clinical variables. These parameters included lymph node status, age, pathohistological grading, tumor size, HER2 status, as well as expression of the LCK metagene. For 124 out of the 334 ER-negative samples and for 37 out of 86 of the ER^+^/HER2^+ ^samples, respectively, all of the parameters were available. As presented in Tables 2 and 3 regarding the ER-negative and the ER^+^/HER2^+ ^samples, respectively, only the LCK metagene remained a significant factor for disease-free survival in this analysis, with a hazard ratio of 2.16 (95% confidence interval = 1.15 to 4.03, *P *= 0.017) and 4.17 (95% confidence interval = 1.38 to 12.6, *P *= 0.011).

**Table 2:** Multivariate Cox regression of lymphocyte-specific kinase metagene and standard parameters among estrogen receptor-negative tumors

Parameter	Stratification	*n*_1_^a^	*n*_2_^a^	*P *value^b^	Hazard ratio	95% confidence interval
Lymphocyte-specific kinase metagene	Low vs. high	60	64	**0.017**	2.16	1.15 to 4.03
Lymph node status	LNN vs. N1	89	35	0.059	1.84	0.98 to 3.47
Age	>50 years vs. ≤ 50 years	71	53	0.91	0.97	0.53 to 1.78
Pathological grading	Poor vs. well/intermediate	83	41	0.97	1.01	0.53 to 1.95
Tumor size	>2 cm vs. ≤ 2 cm	76	48	0.078	1.85	0.93 to 3.66
Human epidermal growth factor receptor 2 status	Positive vs. negative	45	79	0.78	1.09	0.58 to 2.05

^a^Complete information on all parameters was available for 124 estrogen receptor-negative samples. ^b^Significant values presented in bold.

**Table 3:** Multivariate Cox regression of lymphocyte-specific kinase metagene and standard parameters among estrogen receptor-positive HER2-positive tumors

Parameter		*n*_1_^a^	*n*_2_^a^	*P *value^b^	Hazard ratio	95% confidence interval
Lymphocyte-specific kinase metagene	Low vs. high	21	16	**0.011**	4.17	1.38 to 12.6
Lymph node status	LNN vs. N1	24	13	0.438	0.69	0.27 to 1.77
Age	>50 years vs. ≤ 50 years	18	19	0.558	1.38	0.47 to 4.09
Pathological grading	Poor vs. well/intermediate	19	18	0.097	2.27	0.86 to 5.99
Tumor size	>2 cm vs. ≤ 2 cm	15	22	0.405	1.63	0.52 to 5.12

^a^Complete information on all parameters was available for 37 estrogen receptor-positive human epidermal growth factor receptor 2 (HER2)-positive samples. ^b^Significant values presented in bold.

## Discussion

The impact of host factors such as immune cells, stromal environment and chemokines on the development and maintenance of breast cancer has frequently been hypothesized, but still remains a matter of debate (reviewed by Dranoff [49]). In the present study we identified seven clusters of immune-system-related metagenes by large-scale microarray analysis and showed an association with different immunological cell types. The redundant information from several highly correlated ProbeSets allows the construction of robust metagenes that can be used as surrogate markers for the amount of different immune cell types in the breast cancer samples. The relationship of these immunological metagenes with other parameters of the tumors in the combined datasets seems to be complex since no simple associations were found.

High expression of the LCK metagene predicted for better disease-free survival among all subgroups of ER-negative tumors and outperformed all standard parameters in multivariate analysis. Moreover, a positive prognostic value of LCK metagene expression was also observed for those ER-positive tumors with HER2 overexpression. Our results are supported by several other recent studies. Ménard and colleagues have shown that lymphocyte infiltration of breast cancer had a strong positive prognostic value in patients younger than 40 years; however, no association in patients 40 years or older was shown [8]. Although the ER status was not analyzed in their study it is well known that younger age is associated with higher numbers of ER-negative tumors. Alexe and colleagues drew a similar conclusion analyzing only one dataset (Rotterdam dataset, n = 286) [9]. They applied a variety of different clustering procedures to this dataset and proposed to analyze HER2 samples separately. They identified 651 genes among HER2-positive samples by principal component analysis that stratify these samples into two groups. One of the groups was characterized by immune-system-associated genes and improved survival.

Teschendorff and colleagues used three microarray datasets from different platforms and applied a recently developed bioinformatical method to identify subgroups among 186 ER-negative breast cancers. They identified a cluster of ER-negative tumors that display higher expression of six immune-system-related genes and were associated with a better prognosis [50]. Most recently, Finak and colleagues used laser-captured microdissection to analyze the stromal compartment of 53 breast tumors [16]. They identified a good-outcome cluster that was enriched in immune-system-related genes and predicted improved survival in four datasets from different platforms (n = 1,021 total). This cluster contained 22 different genes, 16 of which were also present in our complete immune response cluster. Eight of these 16 genes are included in our LCKmetagene and two genes in our MHC-I metagene.

Calabrò and colleagues, in a computational screening approach to dissect the effect of LI on published ER gene signatures, recently showed that LI is associated with longer survival in ER-negative patients but shorter survival in ER-positive patients [51]. Moreover, Schmidt and colleagues identified B-cell and T-cell metagenes (corresponding to the IgG and LCK metagenes in our study) by hierarchical clustering of 200 untreated breast cancer samples [52]. In contrast to our results with no prognostic value of the B-cell metagene in either ER-positive or ER-negative subgroups (Additional data file 8), these authors identified the B-cell metagene as the most important prognostic factor outperforming the T-cell metagene. Several reasons might account for these discrepancies. Schmidt and colleagues used three patient cohorts all containing only node-negative patients without any adjuvant therapy. Despite these very homogeneous cohorts, in one of them the prognostic value of the B-cell metagene was restricted to the subset of highly proliferating tumors (the discovery cohort Mainz). This specific cohort was characterized by a lower proportion of ER-negative tumors (22%). The study of Schmidt and coworkers clearly demonstrate a prognostic value of lymphocyte metagenes (B-cell and T-cell metagenes). In contrast to the cohorts in the study by Schmidt and colleagues, our sample collective was rather heterogeneous – containing node-positive samples and many patients treated with adjuvant therapy (see Additional data file 2). The difference in our results might therefore be related to different cohorts and treatments as well as a potential predictive value of LI for the response to adjuvant therapy. This possibility could be important since the response rates to neoadjuvant chemotherapy are generally higher for ER-negative tumors [53], the very subgroup in which we observed the prognostic value of the LCK metagene. One hundred and ninety-eight of the samples from our combined datasets were pretherapeutic biopsies from patients treated with neoadjuvant chemotherapy (MDA133 and Frankfurt-2 datasets) [28,29]. In an exploratory analysis, six out of eight samples (75%) with high expression of both IgG and LCK metagenes achieved a pathological complete response – in contrast to only 45 out of all 198 samples (22.7%, *P *= 0.002; Additional data file 9). The ER status, however, might have a confounding effect in this analysis since all six samples were ER-negative. When the samples were further stratified according to the ER status, only a trend to significance (*P *= 0.057) was observed in the ER-negative subgroup. Still, these data suggest that the beneficial effect of the expression of the LCK metagenes in our analyses might at least in part be related to a predictive role in chemotherapeutic treatment.

Despite the observed prognostic and predictive value of LI in our analyses, the molecular mechanism behind this phenomenon is not fully clear. Casares and colleagues have reported that tumor cells dying in response to anthracyclines can induce an antitumor immune response that depends on cytotoxic T cells and dendritic cells [54-56]. These results are in line with the better response to anthracycline-containing neoadjuvant chemotherapy we have observed. While lymphocytes may secrete cytokines resulting in an antitumor response [57], however, they might also shift the balance of the cytokine milieu toward angiogenic factors [58] and inflammatory cytokines that seem to promote tumor progression [59,60]. On the one hand, the tumor-associated lymphocytes might be a marker of an immune response against the tumor. On the other hand, these lymphocytes could be attracted by the tumor cells and generate a functional niche by interaction with the undifferentiated cancer cells. Moreover, whether either modulation of immune response alters the clinical course of breast cancer patients or whether efficacy of specific anticancer treatment approaches depends on the existence of defined tumor host factors and are therefore predictive in some way should be clarified. As a matter of fact it is clear that immune-system-related markers are frequently part of many prognostic and predictive signatures, even though a specific biological role cannot be assigned to date.

## Conclusions

Many prognostic gene signatures has been reported to date that seem to have high rates of concordance in their outcome predictions [61]. In a very recent study, however, Wirapati and coworkers demonstrated that all of these signatures uniformly identify the same group of low-proliferating ER-positive tumors as having a good prognosis [62]. In contrast, all ER-negative tumors are assigned to the poor prognosis group together with high-proliferating ER-positive tumors by all prognostic signatures. An important result of the present work is therefore that the LCK metagene may actually separate the ER-negative group into those tumors with better or worse prognosis.

## Abbreviations

ER: estrogen receptor; HER2: Human epidermal growth factor receptor 2; LCK: lymphocyte specific kinase; LI: lymphocyte infiltration; MHC: major histocompatibility complex; PBS: phosphate-buffered saline; SCL: stem-cell like; STAT1: signal transducer and activator of transcription 1.

## Competing interests

The authors declare that they have no competing interests.

## Authors' contributions

AR, TK and UH conceived the study, carried out the analyses and wrote the manuscript. LP and CL added experimental data, and participated in the interpretation of the data and in writing the manuscript. KE performed pathological examination of samples. RG, ER, LH, AA and CS provided patients and samples, obtained follow-up data and helped to draft the manuscript. DM and TK performed the statistical analysis. MK initiated the study and participated in the design and writing of the manuscript. All authors read and approved the final manuscript.

## Supplementary Material

Additional file 1 An Excel file containing the microarray expression data of the 569 Affymetrix ProbeSets from the immune-related cluster of the datasets Frankfurt and Frankfurt-2 together with estrogen receptor, progesterone receptor, and human epidermal growth factor receptor 2 status (0 = negative, 1 = positive). Click here for file

Additional file 2 An Adobe file containing a table that lists the Affymetrix HG-U133A datasets used in the present study, their usage as well as the characteristics of the respective cohorts. Click here for file

Additional file 3 An Adobe file containing a figure that presents the expression of all 569 Affymetrix ProbeSets from the immune-related gene cluster in a combined cohort of 1,230 samples to tease out their relationship. Click here for file

Additional file 4 An Adobe file containing a table that lists all 569 Affymetrix ProbeSets of the immune-system-related gene cluster from Additional data file 3. Click here for file

Additional file 5 An Adobe file containing a table that presents the detailed list of the 199 Affymetrix ProbeSets of the immune-system-related metagene clusters and their functional annotation. Click here for file

Additional file 6 An Adobe file containing two figures presenting the relationship of the expression of the immune-system-related metagenes with estrogen receptor status, human epidermal growth factor receptor 2 status and the presence of stem-cell like markers in all 1,781 samples from all datasets. Unsupervised clustering analysis of the samples using the immune-related metagenes is presented in Supplemental figure S2a. Scatter plots of the five metagenes representing the major clusters are given in Supplemental figure S2b. Click here for file

Additional file 7 An Adobe file containing a table that presents the results from multivariate Cox regression of immune-system-related metagenes in relation to disease-free survival of breast cancer patients (n = 1,263). Click here for file

Additional file 8 An Adobe file containing a figure that presents a Kaplan–Meier plot that presents the prognostic value of the IgG metagene in breast cancer patients. Click here for file

Additional file 9 An Adobe file containing a figure that presents the relationship of the expression of immune-system-related metagenes and response to neoadjuvant chemotherapy. Pretherapeutic breast cancer samples (n = 198) from patients treated with neoadjuvant chemotherapy were stratified according to the estrogen receptor status of the tumor and were analyzed for expression of IgG and lymphocyte-specific kinase metagenes in a scatter plot. Click here for file

Additional file 10 An Adobe file containing a table that presents a list of the tissue and cell line samples from Figure 2. Gene Expression Omnibus database accession numbers of expression data from Su and colleagues [GEO:GSE1133] [31] and a description of the samples from immunological cell types and tissues as ordered in Figure 2 (left to right) are given. Click here for file

## References

[B1] Coussens LM, Werb Z (2002). Inflammation and cancer. Nature.

[B2] Meylan E, Tschopp J, Karin M (2006). Intracellular pattern recognition receptors in the host response. Nature.

[B3] de Visser KE, Eichten A, Coussens LM (2006). Paradoxical roles of the immune system during cancer development. Nat Rev Cancer.

[B4] Coronella-Wood JA, Hersh EM (2003). Naturally occurring B-cell responses to breast cancer. Cancer Immunol Immunother.

[B5] Wong PY, Staren ED, Tereshkova N, Braun DP (1998). Functional analysis of tumor-infiltrating leukocytes in breast cancer patients. J Surg Res.

[B6] Chin Y, Janseens J, Vandepitte J, Vandenbrande J, Opdebeek L, Raus J (1992). Phenotypic analysis of tumor-infiltrating lymphocytes from human breast cancer. Anticancer Res.

[B7] Kohrt HE, Nouri N, Nowels K, Johnson D, Holmes S, Lee PP (2005). Profile of immune cells in axillary lymph nodes predicts disease-free survival in breast cancer. PLoS Med.

[B8] Ménard S, Tomasic G, Casalini P, Balsari A, Pilotti S, Cascinelli N, Salvadori B, Colnaghi MI, Rilke F (1997). Lymphoid infiltration as a prognostic variable for early-onset breast carcinomas. Clin Cancer Res.

[B9] Alexe G, Dalgin GS, Scanfeld D, Tamayo P, Mesirov JP, DeLisi C, Harris L, Barnard N, Martel M, Levine AJ, Ganesan S, Bhanot G (2007). High expression of lymphocyte-associated genes in node-negative HER2^+ ^breast cancers correlates with lower recurrence rates. Cancer Res.

[B10] Plunkett TA, Correa I, Miles DW, Taylor-Papadimitriou J (2001). Breast cancer and the immune system: opportunities and pitfalls. J Mammary Gland Biol Neoplasia.

[B11] Pupa SM, Ménard S, Andreola S, Colnaghi MI (1993). Antibody response against the c-erbB-2 oncoprotein in breast carcinoma patients. Cancer Res.

[B12] Perou CM, Jeffrey SS, Rijn M van de, Rees CA, Eisen MB, Ross DT, Pergamenschikov A, Williams CF, Zhu SX, Lee JC, Lashkari D, Shalon D, Brown PO, Botstein D (1999). Distinctive gene expression patterns in human mammary epithelial cells and breast cancers. Proc Natl Acad Sci USA.

[B13] Huang E, Cheng SH, Dressman H, Pittman J, Tsou MH, Horng CF, Bild A, Iversen ES, Liao M, Chen CM, West M, Nevins JR, Huang AT (2003). Gene expression predictors of breast cancer outcomes. Lancet.

[B14] Charafe-Jauffret E, Ginestier C, Monville F, Finetti P, Adélaïde J, Cervera N, Fekairi S, Xerri L, Jacquemier J, Birnbaum D, Bertucci F (2006). Gene expression profiling of breast cell lines identifies potential new basal markers. Oncogene.

[B15] Bertucci F, Finetti P, Cervera N, Charafe-Jauffret E, Mamessier E, Adélaïde J, Debono S, Houvenaeghel G, Maraninchi D, Viens P, Charpin C, Jacquemier J, Birnbaum D (2006). Gene expression profiling shows medullary breast cancer is a subgroup of basal breast cancers. Cancer Res.

[B16] Finak G, Bertos N, Pepin F, Sadekova S, Souleimanova M, Zhao H, Chen H, Omeroglu G, Meterissian S, Omeroglu A, Hallett M, Park M (2008). Stromal gene expression predicts clinical outcome in breast cancer. Nat Med.

[B17] Ahr A, Karn T, Solbach C, Seiter T, Strebhardt K, Holtrich U, Kaufmann M (2002). Identification of high risk breast-cancer patients by gene expression profiling. Lancet.

[B18] Rody A, Holtrich U, Gaetje R, Gehrmann M, Engels K, von Minckwitz G, Loibl S, Diallo-Danebrock R, Ruckhäberle E, Metzler D, Ahr A, Solbach C, Karn T, Kaufmann M (2007). Poor outcome in estrogen receptor-positive breast cancers predicted by loss of plexin B1. Clin Cancer Res.

[B19] Miller LD, Smeds J, George J, Vega VB, Vergara L, Ploner A, Pawitan Y, Hall P, Klaar S, Liu ET, Bergh J (2005). An expression signature for p53 status in human breast cancer predicts mutation status, transcriptional effects, and patient survival. Proc Natl Acad Sci USA.

[B20] Sotiriou C, Wirapati P, Loi S, Harris A, Fox S, Smeds J, Nordgren H, Farmer P, Praz V, Haibe-Kains B, Desmedt C, Larsimont D, Cardoso F, Peterse H, Nuyten D, Buyse M, Vijver MJ Van de, Bergh J, Piccart M, Delorenzi M (2006). Gene expression profiling in breast cancer: understanding the molecular basis of histologic grade to improve prognosis. J Natl Cancer Inst.

[B21] Pawitan Y, Bjohle J, Amler L, Borg AL, Egyhazi S, Hall P, Han X, Holmberg L, Huang F, Klaar S, Liu ET, Miller L, Nordgren H, Ploner A, Sandelin K, Shaw PM, Smeds J, Skoog L, Wedren S, Bergh J (2005). Gene expression profiling spares early breast cancer patients from adjuvant therapy: derived and validated in two population-based cohorts. Breast Cancer Res.

[B22] Minn AJ, Gupta GP, Siegel PM, Bos PD, Shu W, Giri DD, Viale A, Olshen AB, Gerald WL, Massagué J (2005). Genes that mediate breast cancer metastasis to lung. Nature.

[B23] Loi S, Haibe-Kains B, Desmedt C, Lallemand F, Tutt AM, Gillet C, Ellis P, Harris A, Bergh J, Foekens JA, Klijn JG, Larsimont D, Buyse M, Bontempi G, Delorenzi M, Piccart MJ, Sotiriou C (2007). Definition of clinically distinct molecular subtypes in estrogen receptor-positive breast carcinomas through genomic grade. J Clin Oncol.

[B24] Wang Y, Klijn JG, Zhang Y, Sieuwerts AM, Look MP, Yang F, Talantov D, Timmermans M, Meijer-van Gelder ME, Yu J, Jatkoe T, Berns EM, Atkins D, Foekens JA (2005). Gene-expression profiles to predict distant metastasis of lymph-node-negative primary breast cancer. Lancet.

[B25] Minn AJ, Gupta GP, Padua D, Bos P, Nguyen DX, Nuyten D, Kreike B, Zhang Y, Wang Y, Ishwaran H, Foekens JA, Vijver M van de, Massagué J (2007). Lung metastasis genes couple breast tumor size and metastatic spread. Proc Natl Acad Sci USA.

[B26] Desmedt C, Piette F, Loi S, Wang Y, Lallemand F, Haibe-Kains B, Viale G, Delorenzi M, Zhang Y, d'Assignies MS, Bergh J, Lidereau R, Ellis P, Harris AL, Klijn JG, Foekens JA, Cardoso F, Piccart MJ, Buyse M, Sotiriou C, TRANSBIG Consortium (2007). Strong time dependence of the 76-gene prognostic signature for node-negative breast cancer patients in the TRANSBIG multicenter independent validation series. Clin Cancer Res.

[B27] International Genomics Consortium Expression Project for Oncology. http://www.intgen.org/.

[B28] Rody A, Karn T, Solbach C, Gaetje R, Munnes M, Kissler S, Ruckhäberle E, Minckwitz GV, Loibl S, Holtrich U, Kaufmann M (2007). The erbB2^+ ^cluster of the intrinsic gene set predicts tumor response of breast cancer patients receiving neoadjuvant chemotherapy with docetaxel, doxorubicin and cyclophosphamide within the GEPARTRIO trial. Breast.

[B29] Hess KR, Anderson K, Symmans WF, Valero V, Ibrahim N, Mejia JA, Booser D, Theriault RL, Buzdar AU, Dempsey PJ, Rouzier R, Sneige N, Ross JS, Vidaurre T, Gómez HL, Hortobagyi GN, Pusztai L (2006). Pharmacogenomic predictor of sensitivity to preoperative chemotherapy with paclitaxel and fluorouracil, doxorubicin, and cyclophosphamide in breast cancer. J Clin Oncol.

[B30] Gene Expression Omnibus. http://www.ncbi.nlm.nih.gov/projects/geo/.

[B31] Su AI, Wiltshire T, Batalov S, Lapp H, Ching KA, Block D, Zhang J, Soden R, Hayakawa M, Kreiman G, Cooke MP, Walker JR, Hogenesch JB (2004). A gene atlas of the mouse and human protein-encoding transcriptomes. Proc Natl Acad Sci USA.

[B32] Affymetrix (2001). Statistical algorithms reference guide Technical report.

[B33] Gautier L, Cope L, Bolstad BM, Irizarry RA (2004). affy – analysis of Affymetrix GeneChip data at the probe level. Bioinformatics.

[B34] Gentleman RC, Carey VJ, Bates DM, Bolstad B, Dettling M, Dudoit S, Ellis B, Gautier L, Ge Y, Gentry J, Hornik K, Hothorn T, Huber W, Iacus S, Irizarry R, Leisch F, Li C, Maechler M, Rossini AJ, Sawitzki G, Smith C, Smyth G, Tierney L, Yang JY, Zhang J (2004). Bioconductor: open software development for computational biology and bioinformatics. Genome Biol.

[B35] Simon R, Radmacher MD, Dobbin K, McShane LM (2003). Pitfalls in the use of DNA microarray data for diagnostic and prognostic classification. J Natl Cancer Inst.

[B36] Rody A, Karn T, Ruckhäberle E, Hanker L, Metzler D, Müller V, Solbach C, Ahr A, Gätje R, Holtrich U, Kaufmann M (2009). Loss of Plexin B1 is highly prognostic in low proliferating ER positive breast cancers – results of a large scale microarray analysis. Eur J Cancer.

[B37] Rody A, Holtrich U, Muller V, Gaetje R, Diallo R, Gehrmann M, von Minckwitz G, Engels K, Karn T, Kaufmann M (2006). c-kit: identification of co-regulated genes by gene expression profiling and clinical relevance of two breast cancer subtypes with stem cell like features. 2006 ASCO Annual Meeting Proceedings Part I. J Clin Oncol.

[B38] Rody A, Karn T, Holtrich U, Kaufmann M (2008). 'Stem cell like' breast cancers – a model for the identification of new prognostic/predictive markers in endocrine responsive breast cancer exemplified by Plexin B1. Eur J Obstet Gynecol Reprod Biol.

[B39] Tsuda H, Morita D, Kimura M, Shinto E, Ohtsuka Y, Matsubara O, Inazawa J, Tamaki K, Mochizuki H, Tamai S, Hiraide H (2005). Correlation of KIT and EGFR overexpression with invasive ductal breast carcinoma of the solid-tubular subtype, nuclear grade 3, and mesenchymal or myoepithelial differentiation. Cancer Sci.

[B40] Symmans WF, Fiterman DJ, Anderson SK, Ayers M, Rouzier R, Dunmire V, Stec J, Valero V, Sneige N, Albarracin C, Wu Y, Ross JS, Wagner P, Theriault RL, Arun B, Kuerer H, Hess KR, Zhang W, Hortobagyi GN, Pusztai L (2005). A single-gene biomarker identifies breast cancers associated with immature cell type and short duration of prior breastfeeding. Endocr Relat Cancer.

[B41] Perou CM, Sorlie T, Eisen MB, Rijn M van de, Jeffrey SS, Rees CA, Pollack JR, Ross DT, Johnsen H, Akslen LA, Fluge O, Pergamenschikov A, Williams C, Zhu SX, Lonning PE, Borresen-Dale AL, Brown PO, Botstein D (2000). Molecular portraits of human breast tumours. Nature.

[B42] Sorlie T, Perou CM, Tibshirani R, Aas T, Geisler S, Johnsen H, Hastie T, Eisen MB, Rijn M van de, Jeffrey SS, Thorsen T, Quist H, Matese JC, Brown PO, Botstein D, Eystein Lonning P, Borresen-Dale AL (2001). Gene expression patterns of breast carcinomas distinguish tumor subclasses with clinical implications. Proc Natl Acad Sci USA.

[B43] Tsuda H, Tani Y, Weisenberger J, Kitada S, Hasegawa T, Murata T, Tamai S, Hirohashi S, Matsubara O, Natori T (2005). Frequent KIT and epidermal growth factor receptor overexpressions in undifferentiated-type breast carcinomas with 'stem-cell-like' features. Cancer Sci.

[B44] R Project for Statistical Computing. http://www.r-project.org.

[B45] Rosen PP, Saigo PE, Braun DW, Weathers E, DePalo A (1981). Predictors of recurrence in stage I (T1N0M0) breast carcinoma. Ann Surg.

[B46] Bilik R, Mor C, Hazaz B, Moroz C (1989). Characterization of T-lymphocyte subpopulations infiltrating primary breast cancer. Cancer Immunol Immunother.

[B47] Ménard S, Casalini P, Tomasic G, Pilotti S, Cascinelli N, Bufalino R, Perrone F, Longhi C, Rilke F, Colnaghi MI (1999). Pathobiologic identification of two distinct breast carcinoma subsets with diverging clinical behaviors. Breast Cancer Res Treat.

[B48] An T, Sood U, Pietruk T, Cummings G, Hashimoto K, Crissman JD (1987). In situ quantitation of inflammatory mononuclear cells in ductal infiltrating breast carcinoma. Relation to prognostic parameters. Am J Pathol.

[B49] Dranoff G (2004). Cytokines in cancer pathogenesis and cancer therapy. Nat Rev Cancer.

[B50] Teschendorff AE, Miremadi A, Pinder SE, Ellis IO, Caldas C (2007). An immune response gene expression module identifies a good prognosis subtype in estrogen receptor negative breast cancer. Genome Biol.

[B51] Calabrò A, Beissbarth T, Kuner R, Stojanov M, Benner A, Asslaber M, Ploner F, Zatloukal K, Samonigg H, Poustka A, Sültmann H (2008). Effects of infiltrating lymphocytes and estrogen receptor on gene expression and prognosis in breast cancer. Breast Cancer Res Treat.

[B52] Schmidt M, Böhm D, von Törne C, Steiner E, Puhl A, Pilch H, Lehr HA, Hengstler JG, Kölbl H, Gehrmann M (2008). The humoral immune system has a key prognostic impact in node-negative breast cancer. Cancer Res.

[B53] Kaufmann M, von Minckwitz G, Rody A (2005). Preoperative (neoadjuvant) systemic treatment of breast cancer. Breast.

[B54] Casares N, Pequignot MO, Tesniere A, Ghiringhelli F, Roux S, Chaput N, Schmitt E, Hamai A, Hervas-Stubbs S, Obeid M, Coutant F, Métivier D, Pichard E, Aucouturier P, Pierron G, Garrido C, Zitvogel L, Kroemer G (2005). Caspase-dependent immunogenicity of doxorubicin-induced tumor cell death. J Exp Med.

[B55] Apetoh L, Ghiringhelli F, Tesniere A, Obeid M, Ortiz C, Criollo A, Mignot G, Maiuri MC, Ullrich E, Saulnier P, Yang H, Amigorena S, Ryffel B, Barrat FJ, Saftig P, Levi F, Lidereau R, Nogues C, Mira JP, Chompret A, Joulin V, Clavel-Chapelon F, Bourhis J, André F, Delaloge S, Tursz T, Kroemer G, Zitvogel L (2007). Toll-like receptor 4-dependent contribution of the immune system to anticancer chemotherapy and radiotherapy. Nat Med.

[B56] Lake RA, Most RG van der (2006). A better way for a cancer cell to die. N Engl J Med.

[B57] Romagnani S (1997). The Th1/Th2 paradigm. Immunol Today.

[B58] Lin EY, Li JF, Gnatovskiy L, Deng Y, Zhu L, Grzesik DA, Qian H, Xue XN, Pollard JW (2006). Macrophages regulate the angiogenic switch in a mouse model of breast cancer. Cancer Res.

[B59] Johansson M, Tan T, de Visser KE, Coussens LM (2007). Immune cells as anti-cancer therapeutic targets and tools. J Cell Biochem.

[B60] Hagemann T, Balkwill F, Lawrence T (2007). Inflammation and cancer: a double-edged sword. Cancer Cell.

[B61] Fan C, Oh DS, Wessels L, Weigelt B, Nuyten DS, Nobel AB, van't Veer LJ, Perou CM (2006). Concordance among gene-expression-based predictors for breast cancer. N Engl J Med.

[B62] Wirapati P, Sotiriou C, Kunkel S, Farmer P, Pradervand S, Haibe-Kains B, Desmedt C, Ignatiadis M, Sengstag T, Schütz F, Goldstein DR, Piccart M, Delorenzi M (2008). Meta-analysis of gene expression profiles in breast cancer: toward a unified understanding of breast cancer subtyping and prognosis signatures. Breast Cancer Res.

