# Supplementary material for: T-cell metagene predicts a favorable prognosis in estrogen receptor-negative and HER2-positive breast cancers
Source: Breast Cancer Res. 2009 Mar 9;11(2):R15. doi: 10.1186/bcr2234 (PMC2688939; doi:10.1186/bcr2234)
Supplement: Additional file 2 — An Adobe file containing a table that lists the Affymetrix HG-U133A datasets used in the present study, their usage as well as the characteristics of the respective cohorts. [file bcr2234-S2.pdf]

Patient characteristics of the Affymetrix HG-U133A datasets used in this study

Dataset	Usage: Metagene identificat.	Usage: Further Usage*	Data Source	No. of samples	% of samples: Age ≤ 50	% of samples: Tumor size ≤ 2 cm	% of samples: LNN	% of samples: ER pos.	% of samples: G3	System. treatment	Median follow up months	No. of relapses	Reference
**Frankfurt**	+	H,C,P	this study	120	54	50	57	66	47	chemotherapy	39	29	Rody et al. 2007a [A]
**Uppsala**	+	C, P	GSE3494	251	22	51	65	80	22	yes / no	118	91	Miller et al. 2005 [B]
**Oxford-Untr.**	+	C, P	GSE2990	61	44	64	100	69	41	untreated	121	29	Sotiriou et al. 2006 [C]
**Stockholm**	+	C, P	GSE1456	159	n.a.	n.a.	n.a.	82	42	yes / no	85	40	Pawitan et al. 2005 [D]
**New York**	+	C, P	GSE2603	99	37	9	34	58	n.a.	n.a.	65	27	Minn et al. 2005 [E]
**London**	+	H, C, P	GSE6532	87	6	35	33	98	23	endocrine	137	28	Loi et al. 2007 [F]
**Rotterdam**	+	C, P	GSE2034, GSE5327	344	n.a.	n.a.	n.a.	61	n.a.	286 untreat., 58 n.a.	86	118	Wang et al. 2005 [G,H]
**Oxford-Tam.**	+	C, P	GSE6532	109	14	34	64	95	19	endocrine	61	30	Loi et al. 2007 [F]
**Villejuif**		C, P	GSE7390	50	80	26	100	72	38	untreated	108	22	Desmedt et al. 2007 [I]
**expO**		C	GSE2109	301	31	32	47	65	49	n.a.	n.a.	n.a.	www.intgen.org [J]
**Frankfurt-2**		C, R	this study	67	51	0	49	58	30	chemotherapy	n.a.	n.a.	Rody et al. 2007b [K]
**MDA133**		C, R	mdanderson.org	133	41	9	30	51	58	chemotherapy	n.a.	n.a.	Hess et al. 2006 [L]
**Total number of samples:**				**1781**	**33**	**33**	**65**	**72**	**37**				
**Cases with follow up information**				**1263**	**31**	**41**	**74**	**74**	**31**		**79**	**395**	

* Further usage of dataset: H=Histological verification, C=Clinical parameter associations, P=Prognostic value, R=Predictive value

[A] Rody A, Holtrich U, Gaetje R, Gehrmann M, Engels K, von Minckwitz G, Loibl S, Diallo-Danebrock R, Ruckhäberle E, Metzler D, Ahr A, Solbach C, Karn T, Kaufmann M. Poor outcome in estrogen receptor-positive breast cancers predicted by loss of plexin B1. Clin Cancer Res. 2007 Feb 15;13(4):1115-22.

[B] Miller LD, Smeds J, George J, Vega VB, Vergara L, Ploner A, Pawitan Y, Hall P, Klaar S, Liu ET, Bergh J. An expression signature for p53 status in human breast cancer predicts mutation status, transcriptional effects, and patient survival. Proc Natl Acad Sci U S A. 2005 Sep 20;102(38):13550-5.

[C] Sotiriou C, Wirapati P, Loi S, Harris A, Fox S, Smeds J, Nordgren H, Farmer P, Praz V, Haibe-Kains B, Desmedt C, Larsimont D, Cardoso F, Peterse H, Nuyten D, Buyse M, Van de Vijver MJ, Bergh J, Piccart M, Delorenzi M. Gene expression profiling in breast cancer: understanding the molecular basis of histologic grade to improve prognosis. J Natl Cancer Inst. 2006 Feb 15;98(4):262-72.

[D] Pawitan Y, Bjohle J, Amler L, Borg AL, Egyhazi S, Hall P, Han X, Holmberg L, Huang F, Klaar S, Liu ET, Miller L, Nordgren H, Ploner A, Sandelin K, Shaw PM, Smeds J, Skoog L, Wedren S, Bergh J. Gene expression profiling spares early breast cancer patients from adjuvant therapy: derived and validated in two population-based cohorts. Breast Cancer Res. 2005;7(6):R953-64.

[E] Minn AJ, Gupta GP, Siegel PM, Bos PD, Shu W, Giri DD, Viale A, Olshen AB, Gerald WL, Massagué J. Genes that mediate breast cancer metastasis to lung. Nature. 2005 Jul 28;436(7050):518-24.

[F] Loi S, Haibe-Kains B, Desmedt C, Lallemand F, Tutt AM, Gillet C, Ellis P, Harris A, Bergh J, Foekens JA, Klijn JG, Larsimont D, Buyse M, Bontempi G, Delorenzi M, Piccart MJ, Sotiriou C. Definition of clinically distinct molecular subtypes in estrogen receptor-positive breast carcinomas through genomic grade. J Clin Oncol. 2007 Apr 1;25(10):1239-46.

[G] Wang Y, Klijn JG, Zhang Y, Sieuwerts AM, Look MP, Yang F, Talantov D, Timmermans M, Meijer-van Gelder ME, Yu J, Jatkoe T, Berns EM, Atkins D, Foekens JA. Gene-expression profiles to predict distant metastasis of lymph-node-negative primary breast cancer. Lancet. 2005; 365(9460):671-9.

[H] Minn AJ, Gupta GP, Padua D, Bos P, Nguyen DX, Nuyten D, Kreike B, Zhang Y, Wang Y, Ishwaran H, Foekens JA, van de Vijver M, Massagué J. Lung metastasis genes couple breast tumor size and metastatic spread. Proc Natl Acad Sci U S A. 2007 Apr 17;104(16):6740-5.

[I] Desmedt C, Piette F, Loi S, Wang Y, Lallemand F, Haibe-Kains B, Viale G, Delorenzi M, Zhang Y, d'Assignies MS, Bergh J, Lidereau R, Ellis P, Harris AL, Klijn JG, Foekens JA, Cardoso F, Piccart MJ, Buyse M, Sotiriou C; TRANSBIG Consortium. Strong time dependence of the 76-gene prognostic signature for node-negative breast cancer patients in the TRANSBIG multicenter independent validation series. Clin Cancer Res. 2007 Jun 1;13(11):3207-14.

[J] The International Genomics Consortium (IGC). The expO project (Expression Project For Oncology) http://www.intgen.org/

[K] Rody A, Karn T, Solbach C, Gaetje R, Munnes M, Kissler S, Ruckhäberle E, Minckwitz GV, Loibl S, Holtrich U, Kaufmann M. The erbB2+ cluster of the intrinsic gene set predicts tumor response of breast cancer patients receiving neoadjuvant chemotherapy with docetaxel, doxorubicin and cyclophosphamide within the GEPARTRIO trial. Breast. 2007 Jun;16(3):235-40.

[L] Hess KR, Anderson K, Symmans WF, Valero V, Ibrahim N, Mejia JA, Booser D, Theriault RL, Buzdar AU, Dempsey PJ, Rouzier R, Sneige N, Ross JS, Vidaurre T, Gómez HL, Hortobagyi GN, Pusztai L. Pharmacogenomic predictor of sensitivity to preoperative chemotherapy with paclitaxel and fluorouracil, doxorubicin, and cyclophosphamide in breast cancer. J Clin Oncol. 2006 Sep 10;24(26):4236-44.
