# Supplementary material for: T-cell metagene predicts a favorable prognosis in estrogen receptor-negative and HER2-positive breast cancers
Source: Breast Cancer Res. 2009 Mar 9;11(2):R15. doi: 10.1186/bcr2234 (PMC2688939; doi:10.1186/bcr2234)
Supplement: Additional file 3 — An Adobe file containing a figure that presents the expression of all 569 Affymetrix ProbeSets from the immune-related gene cluster in a combined cohort of 1,230 samples to tease out their relationship. [file bcr2234-S3.pdf]

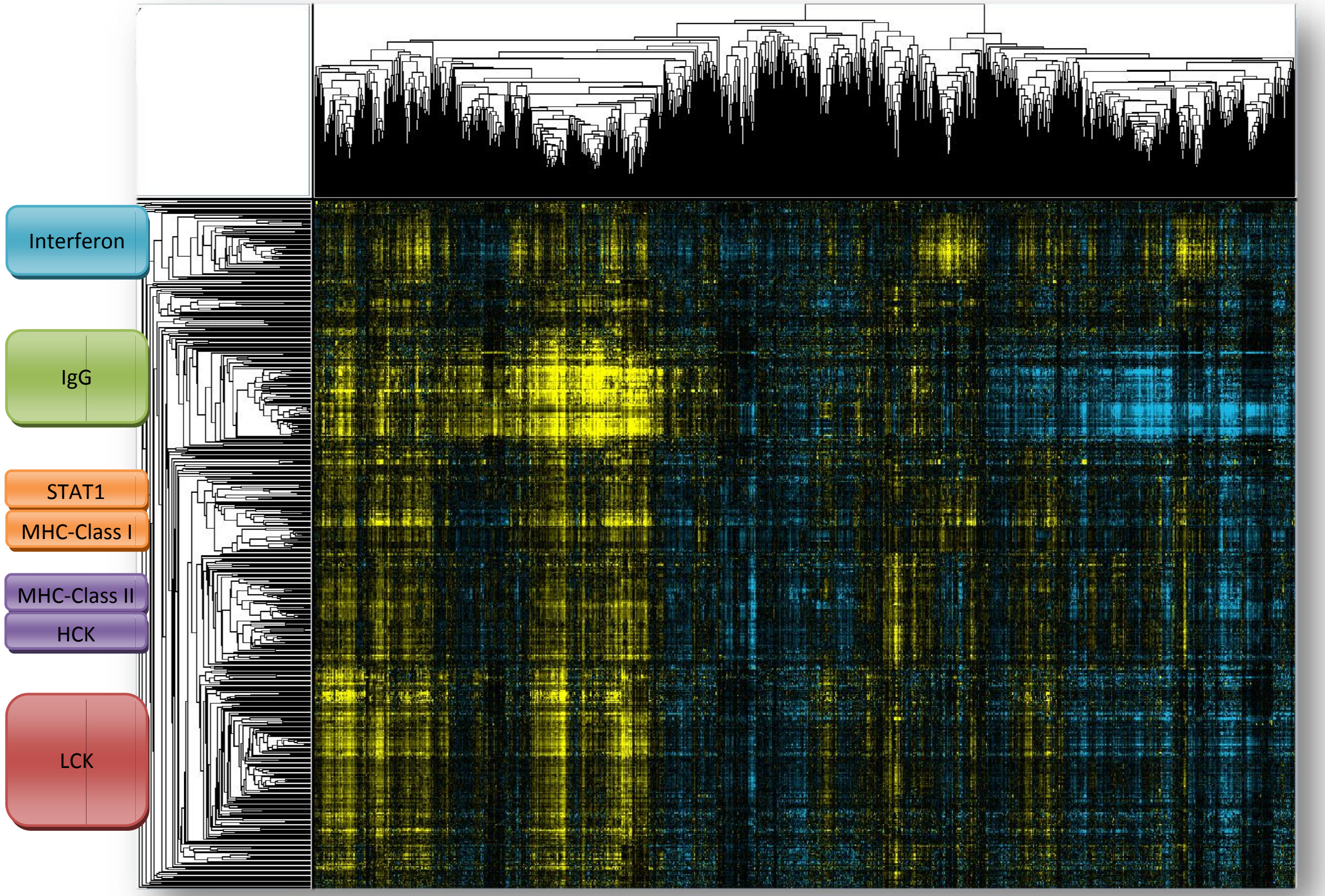

Suppl. Figure 1: The expression of 569 Affymetrix ProbeSets from the immune related gene cluster was analyzed in a combined cohort of n=1230 samples to tease out their relationship. All samples from the following datasets were included in the analysis: Frankfurt, Uppsala, Oxford-Untreated, Oxford-Tam., Stockholm, NewYork, London, Rotterdam (see also Additional data file 2 for information on the datasets).
