# Supplementary material for: T-cell metagene predicts a favorable prognosis in estrogen receptor-negative and HER2-positive breast cancers
Source: Breast Cancer Res. 2009 Mar 9;11(2):R15. doi: 10.1186/bcr2234 (PMC2688939; doi:10.1186/bcr2234)
Supplement: Additional file 4 — An Adobe file containing a table that lists all 569 Affymetrix ProbeSets of the immune-system-related gene cluster from Additional data file 3. [file bcr2234-S4.pdf]

Complete list of the 569 Affymetrix ProbeSets of the immune system related gene cluster from Additional File 2

Affymetrix ProbeSet	Gene Symbol
207794_at	CCR2
207992_s_at	AMPD3
210506_at	FUT7
205312_at	SPI1
206369_s_at	PIK3CG
217369_at	LOC440361
217390_x_at	- - -
202838_at	FUCA1
201288_at	ARHGDIB
203281_s_at	UBE1L
1294_at	UBE1L
216558_x_at	- - -
206313_at	HLA-DOA
207796_x_at	KLRD1
211655_at	IGLJ3
214615_at	P2RY10
204769_s_at	TAP2
217192_s_at	PRDM1
210873_x_at	APOBEC3A
211557_x_at	SLCO2B1
203472_s_at	SLCO2B1
207536_s_at	TNFRSF9
207351_s_at	SH2D2A
216573_at	gene
219403_s_at	HPSE
35150_at	CD40
213038_at	IBRDC3
36564_at	IBRDC3
202074_s_at	OPTN
219574_at	FLJ20668
221060_s_at	TLR4
214560_at	FPRL2
214366_s_at	ALOX5
204445_s_at	ALOX5
203388_at	ARRB2
208130_s_at	TBXAS1
203320_at	LNK
204785_x_at	IFNAR2
211469_s_at	CXCR6
206478_at	KIAA0125
211209_x_at	SH2D1A
220731_s_at	FLJ10420
203927_at	NFKBIE
204224_s_at	GCH1
219424_at	EBI3
211286_x_at	CSF2RA
207085_x_at	CSF2RA
215035_at	IGLV6-57
217084_at	IGHM
217217_at	MGC27165
216846_at	IGLJ3
208602_x_at	CD6
217320_at	- - -
216191_s_at	TRA@
212873_at	HA-1
206687_s_at	PTPN6
202295_s_at	CTSH
210001_s_at	SOCS1
219716_at	APOL6
201641_at	BST2
201762_s_at	PSME2
204804_at	TRIM21
218429_s_at	FLJ11286
53720_at	FLJ11286
217502_at	IFIT2
201315_x_at	IFITM2

Affymetrix ProbeSet	Gene Symbol
216565_x_at	LOC391020
212203_x_at	IFITM3
214022_s_at	IFITM1
201601_x_at	IFITM1
213294_at	- - -
209417_s_at	IFI35
209762_x_at	SP110
208012_x_at	SP110
209761_s_at	SP110
219691_at	SAMD9
201649_at	UBE2L6
203882_at	ISGF3G
219863_at	HERC5
204994_at	MX2
218986_s_at	FLJ20035
219211_at	USP18
219352_at	HERC6
205660_at	OASL
202411_at	IFI27
204415_at	G1P3
202869_at	OAS1
204972_at	OAS2
213797_at	RSAD2
203153_at	IFIT1
214453_s_at	IFI44
204439_at	IFI44L
205483_s_at	G1P2
202086_at	MX1
204747_at	IFIT3
218400_at	OAS3
205552_s_at	OAS1
219209_at	IFIH1
214059_at	IFI44
202446_s_at	PLSCR1
202430_s_at	PLSCR1
218943_s_at	DDX58
218543_s_at	ZC3HDC1
219684_at	IFRG28
210797_s_at	OASL
203595_s_at	IFIT5
203596_s_at	IFIT5
208436_s_at	IRF7
206133_at	HSXIAPAF1
217933_s_at	LAP3
219519_s_at	SN
44673_at	SN
206553_at	OAS2
200628_s_at	WARS
AFFX-HUMISGF3A/M97935_MB_at	- - -
AFFX-HUMISGF3A/M97935_MA_at	- - -
211530_x_at	HLA-G
AFFX-HUMISGF3A/M97935_5_at	- - -
202145_at	LY6E
204858_s_at	ECGF1
200923_at	LGALS3BP
207795_s_at	KLRD1
210865_at	FASLG
222218_s_at	PILRA
204789_at	FMNL1
214551_s_at	CD7
220423_at	PLA2G2D
221210_s_at	NPL
203470_s_at	PLEK
208450_at	LGALS2
204436_at	pp9099
205786_s_at	ITGAM

Affymetrix ProbeSet	Gene Symbol
204446_s_at	ALOX5
206761_at	CD96
221724_s_at	CLEC4A
219947_at	CLEC4A
211144_x_at	TRGV9
216920_s_at	TRGV9
215806_x_at	TRGV9
209813_x_at	TRGV9
214049_x_at	CD7
219593_at	SLC15A3
205685_at	CD86
221004_s_at	ITM2C
218999_at	FLJ11000
209546_s_at	APOL1
212414_s_at	38601
212413_at	38601
221978_at	HLA-F
208966_x_at	IFI16
206332_s_at	IFI16
208965_s_at	IFI16
213982_s_at	RABGAP1L
210176_at	TLR1
204924_at	TLR2
220146_at	TLR7
205859_at	LY86
221581_s_at	WBSCR5
203104_at	CSF1R
211395_x_at	FCGR2C
210992_x_at	FCGR2C
204174_at	ALOX5AP
204006_s_at	FCGR3A
39318_at	TCL1A
209995_s_at	TCL1A
221969_at	PAX5
206060_s_at	PTPN22
216598_s_at	CCL2
214038_at	CCL8
217022_s_at	IGHA2
212592_at	IGJ
217198_x_at	IGH@
216541_x_at	IGHG1
217384_x_at	- - -
205692_s_at	CD38
217360_x_at	IGHG3
216412_x_at	IGLJ3
216542_x_at	MGC27165
216430_x_at	IGLC2
216829_at	IGKC
207734_at	LAX
206181_at	SLAMF1
204562_at	IRF4
215949_x_at	IGHM
209374_s_at	IGHM
211633_x_at	IGHG1
216853_x_at	IGLJ3
216365_x_at	IGLJ3
211430_s_at	IGH@
215214_at	IGLC2
216557_x_at	IGHG1
217227_x_at	IGL@
217258_x_at	IGL
211635_x_at	IGHG3
211634_x_at	IGHM
214973_x_at	IGHD
216491_x_at	IGHM
211637_x_at	LOC388078

Affymetrix ProbeSet	Gene Symbol
217281_x_at	IGHG1
211650_x_at	IGHG1
211908_x_at	IGHG1
216510_x_at	IGHG1
214777_at	IGKC
214768_x_at	IGKC
215121_x_at	IGL@
214677_x_at	IGL@
209138_x_at	IGLC2
221671_x_at	IGKC
221651_x_at	IGKC
214836_x_at	IGKC
214669_x_at	LOC440871
215379_x_at	IGL@
215946_x_at	IGLL1
211643_x_at	IGKC
216401_x_at	- - -
211644_x_at	IGKC
217378_x_at	LOC391427
217157_x_at	IGKC
216207_x_at	IGKV1D-13
217480_x_at	LOC339562
215176_x_at	IGKC
211645_x_at	IGKC
216576_x_at	- - -
216984_x_at	IGLJ3
217148_x_at	IGLC2
211881_x_at	IGLJ3
211798_x_at	IGLJ3
213502_x_at	LOC91316
217179_x_at	IGL@
211868_x_at	IGHG1
217235_x_at	IGLJ3
214916_x_at	IGH@
205267_at	POU2AF1
216560_x_at	IGLC2
211641_x_at	IGHG1
211649_x_at	IGHG1
211639_x_at	IGHM
211640_x_at	IGHG1
206641_at	TNFRSF17
217236_x_at	IGHG1
204269_at	PIM2
205049_s_at	CD79A
221286_s_at	PACAP
216892_at	IGH@
217147_s_at	TCRIM
205718_at	ITGB7
208885_at	LCP1
206398_s_at	CD19
219667_s_at	BANK1
210072_at	CCL19
217418_x_at	MS4A1
210356_x_at	MS4A1
212827_at	IGHM
209829_at	C6orf32
207375_s_at	IL15RA
204153_s_at	MFNG
205038_at	ZNFN1A1
221658_s_at	IL21R
219505_at	CECR1
207957_s_at	PRKCB1
206974_at	CXCR6
216950_s_at	FCGR1A
214511_x_at	FCGR1A
203561_at	FCGR2A

Affymetrix ProbeSet	Gene Symbol
209949_at	NCF2
210889_s_at	FCGR2B
213958_at	CD6
38964_r_at	WAS
204852_s_at	PTPN7
204960_at	PTPRCAP
206513_at	AIM2
213416_at	ITGA4
214219_x_at	MAP4K1
206296_x_at	MAP4K1
205213_at	CENTB1
214450_at	CTSW
204890_s_at	LCK
219812_at	STAG3
219191_s_at	BIN2
206991_s_at	CCR5
202901_x_at	CTSS
204961_s_at	NCF1
214467_at	GPR65
205821_at	KLRK1
203923_s_at	CYBB
207339_s_at	LTB
202665_s_at	WASPIP
213915_at	NKG7
206011_at	CASP1
211367_s_at	CASP1
211368_s_at	CASP1
211366_x_at	CASP1
209970_x_at	CASP1
204205_at	APOBEC3G
204820_s_at	BTN3A3
204821_at	BTN3A3
38241_at	BTN3A3
206134_at	ADAMDEC1
202531_at	IRF1
210029_at	INDO
202270_at	GBP1
202269_x_at	GBP1
204279_at	PSMB9
202307_s_at	TAP1
209969_s_at	STAT1
200887_s_at	STAT1
AFFX-HUMISGF3A/M97935_3_at	- - -
210163_at	CXCL11
211122_s_at	CXCL11
204533_at	CXCL10
203915_at	CXCL9
200629_at	WARS
203547_at	CD4
209606_at	PSCDBP
212587_s_at	PTPRC
200904_at	HLA-E
213566_at	RNASE6
209879_at	SELPLG
204192_at	CD37
203761_at	SLA
209734_at	HEM1
203332_s_at	INPP5D
205270_s_at	LCP2
205159_at	CSF2RB
204774_at	EVI2A
206584_at	LY96
220330_s_at	SAMSN1
216250_s_at	LPXN
204220_at	GMFG
213160_at	DOCK2

Affymetrix ProbeSet	Gene Symbol
206150_at	TNFRSF7
206978_at	CCR2
204661_at	CD52
34210_at	CD52
213603_s_at	RAC2
210031_at	CD3Z
205488_at	GZMA
206337_at	CCR7
218805_at	GIMAP5
64064_at	GIMAP5
209685_s_at	PRKCB1
209083_at	CORO1A
38149_at	KIAA0053
206666_at	GZMK
204118_at	CD48
213539_at	CD3D
205831_at	CD2
213193_x_at	TRBC1
210915_x_at	TRBC1
211796_s_at	TRBC1
204891_s_at	LCK
211339_s_at	ITK
203416_at	CD53
211742_s_at	EVI2B
212588_at	PTPRC
204912_at	IL10RA
205269_at	LCP2
209670_at	TRA@
204655_at	SCYA5
1405_i_at	CCL5
204116_at	IL2RG
204563_at	SELL
204923_at	CXorf9
204057_at	IRF8
202957_at	HCLS1
210972_x_at	TRA@
209671_x_at	TRA@
211902_x_at	TRA@
207238_s_at	PTPRC
219666_at	MS4A6A
209901_x_at	AIF1
213095_x_at	AIF1
215051_x_at	AIF1
211581_x_at	LST1
210629_x_at	LST1
214574_x_at	LST1
214181_x_at	LST1
215633_x_at	LST1
211582_x_at	LST1
201858_s_at	PRG1
218232_at	C1QA
210644_s_at	LAIR1
201720_s_at	LAPTM5
201721_s_at	LAPTM5
202953_at	C1QB
204122_at	TYROBP
204232_at	FCER1G
202803_s_at	ITGB2
204588_s_at	SLC7A7
203473_at	SLCO2B1
212671_s_at	HLA-DQA1
204670_x_at	HLA-DRB1
209619_at	CD74
217478_s_at	HLA-DMA
210982_s_at	HLA-DRA
208894_at	HLA-DRA

Affymetrix ProbeSet	Gene Symbol
211991_s_at	HLA-DPA1
208306_x_at	HLA-DRB1
215193_x_at	HLA-DRB1
209312_x_at	HLA-DRB1
203932_at	HLA-DMB
202902_s_at	CTSS
204834_at	FGL2
204959_at	MNDA
201137_s_at	HLA-DPB1
218870_at	ARHGAP15
213975_s_at	LYZ
201859_at	PRG1
206715_at	TFEC
219243_at	GIMAP4
203471_s_at	PLEK
204882_at	KIAA0053
211795_s_at	FYB
215049_x_at	CD163
203645_s_at	CD163
205098_at	CCR1
219607_s_at	MS4A4A
203508_at	TNFRSF1B
219386_s_at	SLAMF8
210895_s_at	CD86
210785_s_at	C1orf38
207571_x_at	C1orf38
210116_at	SH2D1A
203879_at	PIK3CD
210260_s_at	TNFAIP8
208296_x_at	TNFAIP8
201487_at	CTSC
208018_s_at	HCK
205798_at	IL7R
213888_s_at	T3JAM
219014_at	PLAC8
213293_s_at	TRIM22
210140_at	CST7
206118_at	STAT4
203922_s_at	CYBB
204502_at	SAMHD1
207677_s_at	NCF4
204265_s_at	GPSM3
205147_x_at	NCF4
209040_s_at	PSMB8
217436_x_at	HLA-J
211529_x_at	HLA-G
211528_x_at	HLA-G
211911_x_at	HLA-B
208729_x_at	HLA-B
211799_x_at	HLA-A
221875_x_at	HLA-F
204806_x_at	HLA-F
216526_x_at	HLA-C
209140_x_at	HLA-B
214459_x_at	HLA-C
208812_x_at	HLA-B
215313_x_at	HLA-A
213932_x_at	HLA-A
217456_x_at	HLA-E
200905_x_at	HLA-E
210514_x_at	HLA-G
206082_at	HCP5
216231_s_at	B2M
201422_at	IFI30
214567_s_at	XCL1
203528_at	SEMA4D

Affymetrix ProbeSet	Gene Symbol
203828_s_at	IL32
205456_at	CD3E
207651_at	GPR171
205804_s_at	T3JAM
205495_s_at	GNLY
37145_at	GNLY
214084_x_at	NCF1
209795_at	CD69
205419_at	EBI2
213733_at	MYO1F
209827_s_at	IL16
205504_at	BTK
210784_x_at	LILRB3
210225_x_at	LILRB3
211135_x_at	LILRB3
207104_x_at	LILRB1
211336_x_at	LILRB1
211133_x_at	LILRB3
206219_s_at	VAV1
203760_s_at	SLA
202659_at	PSMB10
217143_s_at	TRA@
202910_s_at	CD97
205992_s_at	IL15
211005_at	LAT
203868_s_at	VCAM1
214054_at	DOK2
210279_at	GPR18
219183_s_at	PSCD4
205758_at	CD8A
214470_at	KLRB1
208146_s_at	CPVL
214339_s_at	MAP4K1
207777_s_at	SP140
220577_at	FLJ13373
206804_at	CD3G
214032_at	ZAP70
210321_at	GZMH
210606_x_at	KLRD1
204929_s_at	VAMP5
210439_at	ICOS
205484_at	SIT
219690_at	FLJ22573
209770_at	BTN3A1
202693_s_at	STK17A
206206_at	LY64
210448_s_at	P2RX5
219118_at	FKBP11
219117_s_at	FKBP11
221253_s_at	TXNDC5
207697_x_at	LILRB2
205997_at	ADAM28
211178_s_at	PSTPIP1
210038_at	PRKCQ
207419_s_at	RAC2
206486_at	LAG3
220059_at	BRDG1
221698_s_at	CLEC7A
203402_at	KCNAB2
202748_at	GBP2
219159_s_at	SLAMF7
219279_at	DOCK10
207540_s_at	SYK
205291_at	IL2RB
205686_s_at	CD86
213830_at	TRD@

**Affymetrix ProbeSet**	**Gene Symbol**
219971_at	IL21R
220418_at	UBASH3A
211211_x_at	SH2D1A
205099_s_at	CCR1
205039_s_at	ZNFN1A1
212415_at	38601
211647_x_at	IGHG1
209166_s_at	MAN2B1
202638_s_at	ICAM1
202637_s_at	ICAM1
204513_s_at	ELMO1
217549_at	- - -
208594_x_at	LILRB6
208071_s_at	LAIR1
205285_s_at	FYB
214228_x_at	TNFRSF4
220132_s_at	CLEC2D
205885_s_at	ITGA4
208438_s_at	FGR
214186_s_at	37106
210786_s_at	FLI1
206589_at	GFI1
207610_s_at	EMR2
211661_x_at	PTAFR
214995_s_at	APOBEC3G
211434_s_at	CCRL2
209124_at	MYD88
215524_x_at	TRA@
221218_s_at	TPK1
208829_at	TAPBP
217992_s_at	EFHD2
220005_at	P2RY13
221331_x_at	CTLA4
207224_s_at	SIGLEC7
219202_at	FLJ22341
209880_s_at	SELPLG
211013_x_at	PML
206503_x_at	PML
220832_at	TLR8
201089_at	ATP6V1B2
214847_s_at	GPSM3
