# Supplementary material for: T-cell metagene predicts a favorable prognosis in estrogen receptor-negative and HER2-positive breast cancers
Source: Breast Cancer Res. 2009 Mar 9;11(2):R15. doi: 10.1186/bcr2234 (PMC2688939; doi:10.1186/bcr2234)
Supplement: Additional file 5 — An Adobe file containing a table that presents the detailed list of the 199 Affymetrix ProbeSets of the immune-system-related metagene clusters and their functional annotation. [file bcr2234-S5.pdf]

Detailed list of the 199 Affymetrix ProbeSets of the immune system related “metagene clusters” and their functional annotation.

Metagene Cluster	Affy_ID	Gene Symbol	Function / cell type	Description
**IgG**	205267_at	POU2AF1	B cell transcription factor	oct-binding factor POU domain class 2 associating factor 1 oct-binding factor POU domain, class 2, associating factor 1
**IgG**	209138_x_at	IGLC2	Immunoglobulin	rearranged immunoglobulin lambda light chain (hybridoma H210) anti-hepatitis A immunoglobulin lambda chain variable region constant region complementarity-determining regions d immunoglobulin lambda locus Ig lambda light chain; immunoglobulin
**IgG**	209374_s_at	IGHM	Immunoglobulin	IgM heavy chain constant region (Ab63) immunoglobulin heavy constant mu
**IgG**	211430_s_at	IGH@	Immunoglobulin	immunoglobulin lambda heavy chain (hybridoma H210) anti-hepatitis A IgG variable region constant region complementarity-determining regions d immunoglobulin heavy constant gamma 3 (Gm marker) constant region; IgG1; immunoglobulin; lambda heavy chain; variable region immunoglobulin lambda heavy chain immunoglobulin heavy constant gamma 3 (G3m marker)
**IgG**	211633_x_at	IGHG1	Immunoglobulin	Ig rearranged H-chain V-region (C-D-JH4)d
**IgG**	211634_x_at	IGHM	Immunoglobulin	Ig rearranged H-chain V-region (C-D-JH6)d
**IgG**	211635_x_at	IGHG3	Immunoglobulin	Ig rearranged H-chain V-region (C-D-JH6)d
**IgG**	211637_x_at	LOC388078	Immunoglobulin	Ig rearranged gamma-chain V-DXP4-JH6cd
**IgG**	211639_x_at	IGHM	Immunoglobulin	oo67b04.s1 Ig rearranged gamma-chain V-DXP1-JH4bd Human anti-B cell autoantibody IgM heavy chain variable V-D-J region (VH4) gene clone E11 VH4-63 non-productive rearrangement EST
**IgG**	211640_x_at	IGHG1	Immunoglobulin	Ig rearranged gamma-chain V-DK4-JH4bd
**IgG**	211641_x_at	IGHG1	Immunoglobulin	IG VH-region gened
**IgG**	211643_x_at	IGKC	Immunoglobulin	Ig rearranged kappa-chain gene V-J-regiond
**IgG**	211644_x_at	IGKC	Immunoglobulin	Ig rearranged kappa-chain gene V-J-regiond
**IgG**	211645_x_at	IGKC	Immunoglobulin	immunoglobulin kappa-chain VK-1 (IgK) d
**IgG**	211649_x_at	IGHG1	Immunoglobulin	wc37g12.x1 Ig rearranged mu-chain gene V-N-D-N-J-regiond integrin cytoplasmic domain-associated protein 1 EST
**IgG**	211650_x_at	IGHG1	Immunoglobulin	IgG heavy chain variable region (Vh26) Ig rearranged mu-chain gene VH3-D2110-JH2ds. polyreactiv Human rearranged immunoglobulin heavy chain mRNA partial cds IgG heavy chain variable region
**IgG**	211798_x_at	IGLJ3	Immunoglobulin	rearranged immunoglobulin lambda light chain for single-chain antibodyd immunoglobulin lambda locus Ig lambda light chain; immunoglobulin immunoglobulin lambda joining 3
**IgG**	211868_x_at	IGHG1	Immunoglobulin	for single-chain antibodyds
**IgG**	211881_x_at	IGLJ3	Immunoglobulin	rearranged immunoglobulin lambda light chain for VEGF single chain antibodyd immunoglobulin lambda locus Ig lambda light chain; immunoglobulin immunoglobulin lambda joining 3
**IgG**	211908_x_at	IGHG1	Immunoglobulin	IgG heavy chain variable region (Vh26) rearranged immunoglobulin heavy chain partial Human rearranged immunoglobulin heavy chain mRNA partial cds IgG heavy chain variable region
**IgG**	213502_x_at	LOC91316	Immunoglobulin	wo05c02.x1 immunoglobulin lambda-like polypeptide 3
**IgG**	214669_x_at	LOC440871	Immunoglobulin	Ig rearranged gamma chainV-J-C region and immunoglobulin kappa variable 3D-15 immunoglobulin kappa constant
**IgG**	214677_x_at	IGL@	Immunoglobulin	rearranged immunoglobulin lambda light chain rearranged immunoglobulin lambda light chain mRN immunoglobulin lambda locus Ig lambda light chain; immunoglobulin immunoglobulin lambda joining 3
**IgG**	214768_x_at	IGKC	Immunoglobulin	active IgK chain from GM 607 V-kappa-2 region
**IgG**	214777_at	IGKC	Immunoglobulin	rearranged gene for kappa immunoglobulin subgroup V kappa IV
**IgG**	214836_x_at	IGKC	Immunoglobulin	Ig rearranged gamma chainV-J-C region and kappa-immunoglobulin germline pseudogene (Chr22.4) variable region (subgroup V kappa II) immunoglobulin kappa constant
**IgG**	214916_x_at	IGH@	Immunoglobulin	IgG heavy chain variable region (Vh26) rearranged immunoglobulin heavy chain ds IgG heavy chain variable region
**IgG**	214973_x_at	IGHD	Immunoglobulin	partial IGVH3 gene for immunoglobulin heavy chain V region case 2 cell E 172
**IgG**	215121_x_at	IGL@	Immunoglobulin	rearranged immunoglobulin lambda light chain immunoglobulin lambda locus Ig lambda light chain; immunoglobulin
**IgG**	215176_x_at	IGKC	Immunoglobulin	partial IGKV gene for immunoglobulin kappa chain variable region clone 30
**IgG**	215214_at	IGLC2	Immunoglobulin	clone ASPBLL54 immunoglobulin lambda light chain VJ region clone ASPBLL54 immunoglobulin lambda light chain VJ region ds Homo sapiens clone ASPBLL54 immunoglobulin lambda light chain VJ region mRNA partial cds truncated protein immunoglobulin lambda light chain VJ region
**IgG**	215379_x_at	IGL@	Immunoglobulin	rearranged immunoglobulin lambda light chain immunoglobulin lambda joining 3 immunoglobulin lambda locus Ig lambda light chain; immunoglobulin
**IgG**	215946_x_at	IGLL1	Immunoglobulin	wo05c02.x1 DNA sequence from clone CTA-246H3 on chromosome 22 Contains the gene for IGLL1 (immunoglobulin lambda-like polypeptide 1 pre-B-cell specific) a pseudogene similar to LRP5 (Lipoprotein Receptor Related Protein.) ESTs Genomic markers (D22S. immunoglobulin lambda-like polypeptide 3
**IgG**	215949_x_at	IGHM	Immunoglobulin	HUMCSFGMA granulocyte-macrophage colony-stimulating factor (CSF1) gene colony stimulating factor 2 (granulocyte-macrophage) granulocyte-macrophage colony stimulating factor granulocyte-macrophage colony-stimulating factor
**IgG**	216207_x_at	IGKV1D-13	Immunoglobulin	germ line pseudogeneimmunoglobulin kappa light chain leader peptide and variable region (subgroup V kappa I) immunoglobulin kappa variable 1-13 germ line; Ig light chain; immunoglobulin; pseudogene; signal peptide; variable region immunoglobulin kappa light chain subgroup V
**IgG**	216365_x_at	IGLJ3	Immunoglobulin	clone bsmneg3-t7 immunoglobulin lambda light chain VJ region (IGL) ds
**IgG**	216401_x_at	- - -	Immunoglobulin	partial IGKV gene for immunoglobulin kappa chain variable region clone 38
**IgG**	216491_x_at	IGHM	Immunoglobulin	immunoglobulin heavy chain variable region (V4-31) gene immunoglobulin heavy chain variable region (V4-4) geneds Homo sapiens partial VH4-30 gene for rearranged immunoglobulin heavy chain variable region isolate case2-Ki67--158 Ig VH4 heavy chain immunoglobulin heavy chain variable region
**IgG**	216510_x_at	IGHG1	Immunoglobulin	IgG heavy chain variable region (Vh26) rearranged immunoglobulin heavy chain ds IgG heavy chain variable region
**IgG**	216557_x_at	IGHG1	Immunoglobulin	rearranged immunoglobulin heavy chain (A1VH3) gene
**IgG**	216560_x_at	IGLC2	Immunoglobulin	immunoglobulin lambda gene locus DNA clone:84 immunoglobulin lambda variable 3-10
**IgG**	216576_x_at	- - -	Immunoglobulin	isolate donor N clone N88K immunoglobulin kappa light chain variable region ds
**IgG**	216853_x_at	IGLJ3	Immunoglobulin	clone KM36 immunoglobulin light chain variable region ds

Metagene Cluster	Affy_ID	Gene Symbol	Function / cell type	Description
IgG	216984_x_at	IGLJ3	Immunoglobulin	immunoglobulin (mAb59) light chain V region sequence immunoglobulin (mAb59) light chain V region partial sequence immunoglobulin light chain V region immunoglobulin light chain V-J region
IgG	217148_x_at	IGLC2	Immunoglobulin	anti-streptococcalanti-myosin immunoglobulin lambda light chain variable region ds
IgG	217157_x_at	IGKC	Immunoglobulin	isolate donor N clone N8K immunoglobulin kappa light chain variable region ds
IgG	217179_x_at	IGL@	Immunoglobulin	(T1.1) for IG lambda light chain
IgG	217227_x_at	IGL@	Immunoglobulin	for IgG lambda light chain V-J-C region (clone Tgl11)
IgG	217235_x_at	IGLJ3	Immunoglobulin	immunoglobulin (mAb56) light chain V region partial sequence
IgG	217258_x_at	IGL	Immunoglobulin	IgG lambda light chain V-J-C region (clone Tgl4) clone ASMneg1-b3 immunoglobulin lambda chain VJ region (IGL) ds H.sapiens mRNA for IgG lambda light chain V-J-C region (clone Tgl9) constant region; immunoglobulin; immunoglobulin lambda chain; immunoglobulin light chain; joining region; variable region immunoglobulin lambda light chain VJC region
IgG	217281_x_at	IGHG1	Immunoglobulin	for immunoglobulin heavy chain variable region ID 3
IgG	217378_x_at	LOC391427	Immunoglobulin	V108 gene encoding an immunoglobulin kappa orph immunoglobulin kappa variable 1/OR2-108
IgG	217480_x_at	LOC339562	Immunoglobulin	kappa-immunoglobulin germline pseudogene (cos118) variable region (subgroup V kappa I) kappa-immunoglobulin germline pseudogene (cos118) variable region (subgroup V kappa I) immunoglobulin kappa variable 1/OR15-118 C-region; V-region; immunoglobulin-kappa; immunoglobulin-kappa subgroup vk-1; orphon; pseudogene Ig kappa chain
IgG	221651_x_at	IGKC	Immunoglobulin	Ig rearranged gamma chainV-J-C region and Similar to immunoglobulin kappa constant clone MGC:124 immunoglobulin kappa constant
IgG	221671_x_at	IGKC	Immunoglobulin	Ig rearranged gamma chainV-J-C region and Ig rearranged gamma chain V-J-C region and complete cd immunoglobulin kappa constant
HCK	201422_at	IFI30	IFN gamma response	HUMIIP gamma-interferon-inducible protein (IP-30) interferon gamma-inducible protein 30 interferon, gamma-inducible protein 30
HCK	201720_s_at	LAPTM5	hematopoietic cells	lysosomal-associated multitransmembrane protein (LAPTm5) Lysosomal-associated multispanning membrane protein-5 Lysosomal-associated multispanning membrane protein-5
HCK	201721_s_at	LAPTM5	hematopoietic cells	lysosomal-associated multitransmembrane protein (LAPTm5) Lysosomal-associated multispanning membrane protein-5 Lysosomal-associated multispanning membrane protein-5
HCK	202803_s_at	ITGB2	subunit of complement receptors	leukocyte adhesion protein (LFA-1 Mac-1 p150 95 family) beta subunit integrin beta 2 (antigen CD18 (p95) lymphocyte function-associated antigen 1 macrophage antigen 1 (mac-1) beta subunit) cell adhesion molecule; cell surface glycoprotein; glycoprotein; leukocyte adhesion protein leukocyte adhesion protein beta-subunit precursor integrin beta chain, beta 2 precursor integrin, beta 2 (antigen CD18 (p95), lymphocyte function-associated
HCK	202953_at	C1QB	complement system	C1q B-chain of complement system complement component 1 q subcomponent betapolypeptide precursor complement component 1 q subcomponent beta polypeptide complement component 1, q subcomponent, beta polypeptide
HCK	203473_at	SLCO2B1	-	KIAA0880 protein solute carrier family 21 (organic anion transporter) member 9 solute carrier family 21 (organic anion transporter), member 9
HCK	203645_s_at	CD163	macrophages	M130 antigen extracellular variant CD163 antigen antigen; antigen M130 M130 antigen extracellular variant
HCK	204122_at	TYROBP	monocyte / myeloid / dendritic cells	zd27g05.s1 TYRO protein tyrosine kinase binding protein
HCK	204232_at	FCER1G	FCeR (mast cells, basophiles)	Fc-epsilon-receptor gamma-chain Fc fragment of IgE high affinity I receptorfor gamma polypeptide precursor Fc fragment of IgE high affinity I receptor for; gamma polypeptide Fc-epsilon-receptor gamma-chain protein Fc-epsilon-receptor gamma-chain protein precursor Fc fragment of IgE, high affinity I, receptor for, gamma polypeptide precursor Fc fragment of IgE, high affinity I, receptor for; gamma
HCK	204588_s_at	SLC7A7	-	glycoprotein-associated amino acid transporter y+LAT1 solute carrier family 7 (cationic amino acid transporter y+ system) member 7 glycoprotein-associated amino acid transporter; y+LAT1 gene glycoprotein-associated amino acid transporter solute carrier family 7 (cationic amino acid transporter,
HCK	205098_at	CCR1	macrophages	HM145 chemokine (C-C motif) receptor 1
HCK	206715_at	TFEC	monocyte / myeloid / dendritic cells	TFEC isoform (or TFECL) transcription factor EC TFEC TFEC isoform (or TFECL)
HCK	208018_s_at	HCK	monocyte / myeloid / dendritic cells	hemopoietic cell protein-tyrosine kinase (HCK) gene clone lambda-a2 1a hemopoietic cell kinase
HCK	209949_at	NCF2	monocyte / myeloid / dendritic cells	neutrophil oxidase factor (p67-phox) Similar to neutrophil cytosolic factor 2 (65kD chronic granulomatous disease autosomal 2) clone MGC:22 neutrophil cytosolic factor 2 (65kD chronic granulomatous disease autosomal 2) neutrophil cytosolic factor 2 (65kD, chronic granulomatous
HCK	210644_s_at	LAIR1	myeloid / T / B cells	leukocyte-associated Ig-like receptor-1 (LAIR-1) leukocyte-associated Ig-like receptor 1b d leukocyte-associated Ig-like receptor 1 membrane glycoprotein leukocyte-associated Ig-like receptor 1
HCK	210895_s_at	CD86	B7-2 costimulatory molecule of APCs	om19c02.s1 CTLA4 counter-receptor (B7-2) d ESTs CD86 antigen (CD28 antigen ligand 2, B7-2 antigen)
HCK	215049_x_at	CD163	macrophages	M130 antigen cytoplasmic variant 2 for M130 antigen cytoplasmic variant CD163 antigen antigen; antigen M130 CD163 antigen
HCK	218232_at	C1QA	complement system	ws52e08.x1 complement component 1 q subcomponent alphapolypeptide precursor complement component 1 q subcomponent alpha polypeptide EST complement component 1, q subcomponent, alpha polypeptide
HCK	219607_s_at	MS4A4A	-	membrane-spanning 4-domains subfamily A member 4 membrane-spanning 4-domains, subfamily A, member 4
HCK	204959_at	MNDA	IFN gamma response	myeloid cell nuclear differentiation antigen myeloid cell nuclear differentiation antigen interferon response element; interferon stimulated gene; interferon-alpha; myeloid cell nuclear differentiation antigen myeloid cell nuclear differentiation antigen
HCK	209901_x_at	AIF1	IFN gamma response	allograft inflammatory factor-1 (AIF-1) allograft-inflammatory factor-1 d allograft inflammatory factor 1 interferon gamma responsive transcript

Metagene Cluster	Affy_ID	Gene Symbol	Function / cell type	Description
HCK	210629_x_at	LST1	monocyte / myeloid / dendritic cells	LST1cLST1 C splice variant LST1 cLST1A splice variantd DNA segment on chromosome 6 (unique) 49 expressed sequence cLST1/C splice variant DNA segment on chromosome 6 (unique) 49 expressed sequence lymphocyte antigen 117
HCK	211581_x_at	LST1	monocyte / myeloid / dendritic cells	LST1cLST1 C splice variant LST1 cLST1E splice variantd DNA segment on chromosome 6 (unique) 49 expressed sequence cLST1/C splice variant DNA segment on chromosome 6 (unique) 49 expressed sequence lymphocyte antigen 117
HCK	211582_x_at	LST1	monocyte / myeloid / dendritic cells	LST1cLST1 C splice variant LST1 cLST1C splice variantd DNA segment on chromosome 6 (unique) 49 expressed sequence cLST1/C splice variant DNA segment on chromosome 6 (unique) 49 expressed sequence lymphocyte antigen 117
HCK	213095_x_at	AIF1	IFN gamma response	allograft inflammatory factor-1 (AIF-1) allograft inflammatory factor-1 splice variant G1 d allograft inflammatory factor 1 interferon gamma responsive transcript
HCK	213160_at	DOCK2	leucocytes	KIAA0209 gene for KIAA0209 gened dedicator of cyto-kinesis 2 KIAA0209 similar to a human major CRK-binding protein DOCK180.
HCK	213566_at	RNASE6	-	qb47d08.x1 ribonuclease RNase A family k6 EST ribonuclease, RNase A family, k6
HCK	214181_x_at	LST1	monocyte / myeloid / dendritic cells	LST1cLST1 C splice variant lymphocyte antigen 117 DNA segment on chromosome 6 (unique) 49 expressed sequence cLST1/C splice variant DNA segment on chromosome 6 (unique) 49 expressed sequence
HCK	214574_x_at	LST1	monocyte / myeloid / dendritic cells	LST1cLST1 C splice variant DNA segment on chromosome 6 (unique) 49 expressed sequence NK cell triggering receptor p30 DNA segment on chromosome 6 (unique) 49 expressed sequence cLST1/C splice variant DNA segment on chromosome 6 (unique) 49 expressed sequence lymphocyte antigen 117
HCK	215051_x_at	AIF1	IFN gamma response	allograft inflammatory factor-1 (AIF-1) allograft inflammatory factor 1 interferon gamma responsive transcript
HCK	215633_x_at	LST1	monocyte / myeloid / dendritic cells	LST1cLST1 C splice variant for LST-1N protein DNA segment on chromosome 6 (unique) 49 expressed sequence cLST1/C splice variant DNA segment on chromosome 6 (unique) 49 expressed sequence lymphocyte antigen 117
HCK	219666_at	MS4A6A	-	KAIA2674 membrane-spanning 4-domains, subfamily A, member 6A
MHC-II	201137_s_at	HLA-DPB1	MHC II component	MHC class II lymphocyte antigen (HLA-DP) beta chain major histocompatibility complex class II DP beta 1 major histocompatibility complex, class II, DP beta 1
MHC-II	201858_s_at	PRG1	-	hematopoetic proteoglycan core protein secretory granule proteoglycan peptide core d proteoglycan 1 secretory granule haematopoetic proteoglycan core protein hematopoetic proteoglycan core protein (AA 1 - 158) proteoglycan 1, secretory granule
MHC-II	202902_s_at	CTSS	MHC II pathway peptidase	wg62c06.x1 cathepsin S
MHC-II	203932_at	HLA-DMB	MHC II component	HLA-DMB major histocompatibility complex class II DM beta major histocompatibility complex, class II, DM beta
MHC-II	204670_x_at	HLA-DRB1	MHC II component	MHC class II HLA-DR beta-1 (DR2.3) 5end major histocompatibility complex class II DR beta 5 major histocompatibility complex class II DR beta 1 major histocompatibility complex, class II, DR beta 5
MHC-II	205270_s_at	LCP2	T-cell activation	76 kDa tyrosine phosphoprotein SLP-76 lymphocyte cytosolic protein 2 (SH2 domain-containing leukocyte protein of 76kD)
MHC-II	207238_s_at	PTPRC	CD45 / leukocyte common antigen	leukocyte common antigen (T200) protein tyrosine phosphatase receptor type C protein tyrosine phosphatase, receptor type, C
MHC-II	208306_x_at	HLA-DRB1	MHC II component	MHC class II HLA-DR beta-1 (DR2.3) 5end major histocompatibility complex class II DR beta 4 major histocompatibility complex class II DR beta 1 major histocompatibility complex, class II, DR beta 4
MHC-II	208894_at	HLA-DRA	MHC II component	major histocompatibility complex class II DR alpha major histocompatibility complex, class II, DR alpha
MHC-II	209312_x_at	HLA-DRB1	MHC II component	MHC class II HLA-DR beta-1 (DR2.3) 5end MHC class II antigen (HLA-DRB1) HLA-DRB1*PBL alleled major histocompatibility complex class II DR beta 1 major histocompatibility complex, class II, DR beta 1
MHC-II	209619_at	CD74	MHC II component	Ia-associated invariant gamma-chain gene CD74 antigen (invariant polypeptide of major histocompatibility complex class II antigen-associated) Ia-associated gamma chain; cell surface glycoprotein Ia-associated gamma chain cell surface glycoprotein
MHC-II	210982_s_at	HLA-DRA	MHC II component	major histocompatibility complex class II DR alpha
MHC-II	211991_s_at	HLA-DPA1	MHC II component	SB classII histocompatibility antigen alpha-chain for SB classII histocompatibility antigen alpha-chain major histocompatibility complex class II DP alpha 1 major histocompatibility complex, class II, DP alpha
MHC-II	212671_s_at	HLA-DQA1	MHC II component	ak41e04.s1 major histocompatibility complex class II DQ alpha 1 EST
MHC-II	215193_x_at	HLA-DRB1	MHC II component	oc35c12.s1 for MHC class II antigen (HLA-DRB1 gene) DRB1*0402 allel ESTs
MHC-II	217478_s_at	HLA-DMA	MHC II component	RING6 HLA class II alpha chain-like product major histocompatibility complex class II DM alpha HLA class II antigen HLA class II alpha chain-like major histocompatibility complex, class II, DM alpha
LCK	1405_i_at	CCL5	T-cell / NK-cell	small inducible cytokine A5 (RANTES)
LCK	204116_at	IL2RG	IL2-Receptor (T-, NK-, B-cells)	HUMIL2RG interleukin 2 receptor gamma chain interleukin 2 receptor gamma (severe combined immunodeficiency) interleukin 2 receptor; interleukin 2 receptor gamma chain interleukin 2 receptor gamma chain interleukin 2 receptor, gamma (severe combined immunodeficiency)
LCK	204118_at	CD48	T-cell / B-cell / NK-cell	MEM-102 glycoprotein CD48 antigen (B-cell membrane protein) MEM-102 glycoprotein; leukocyte antigen CD48 antigen (B-cell membrane protein)
LCK	204563_at	SELL	L-selectin (leukocyte homing)	HUMLNHR lymph node homing receptor selectin L (lymphocyte adhesion molecule 1) lymph node homing receptor precursor selectin L
LCK	204655_at	SCYA5	T-cell specific (RANTES)	HUMTCSM T cell-specific protein (RANTES) small inducible cytokine A5 (RANTES) Alu repeat; T-cell-specific protein T cell-specific protein precursor small inducible cytokine A5 (RANTES)

Metagene Cluster	Affy_ID	Gene Symbol	Function / cell type	Description
**LCK**	204891_s_at	LCK	T-cell specific	T-lymphocyte specific protein tyrosine kinase p56lck (lck) abberant lymphocyte-specific protein tyrosine kinase truncated form of T-lymphocyte-specific protein tyrosine kinase p56lck; this abberant message encoding primarily the SH2 and SH3 domains of p56lck was observed by nothern hybridization and PCR amplification in poly-A selected RNA from two human leukemic T p56lck
**LCK**	205488_at	GZMA	T-cell	Hanukah factor serine protease (HuHF) granzyme A (granzyme 1 cytotoxic T-lymphocyte-associated serine esterase 3) granzyme A (granzyme 1, cytotoxic T-lymphocyte-associated
**LCK**	205798_at	IL7R	IL7-Receptor	HUMIL7AA interleukin-7 receptor (IL-7) interleukin 7 receptor interleukin 7 receptor interleukin-7 receptor precursor interleukin 7 receptor
**LCK**	205821_at	KLRK1	NK-cell	NKG2D gene exons 2-5 and joined and CDS DNA segment on chromosome 12 (unique) 2489 expressed sequence NKG2 gene; NKG2D gene DNA segment on chromosome 12 (unique) 2489 expressed sequence
**LCK**	205831_at	CD2	T-cell	T-cell surface antigen CD2 (T11) clone PB1 CD2 antigen (p50) sheep red blood cell receptor CD2 antigen (p50), sheep red blood cell receptor
**LCK**	206118_at	STAT4	T-cell differentiation	HUMSTAT4R STAT4 signal transducer and activator of transcription 4
**LCK**	206150_at	TNFRSF7	T-cell activation	T cell activation antigen (CD27) tumor necrosis factor receptor superfamily member 7 tumor necrosis factor receptor superfamily, member 7
**LCK**	206181_at	SLAMF1	B-cell / T-cell	signaling lymphocytic activation molecule (SLAM) signaling lymphocytic activation molecule signaling lymphocytic activation molecule
**LCK**	206337_at	CCR7	T-cell / B-cell chemokine receptor	HUMEBI103 G protein-coupled receptor (EBI 1) gene exon 3 chemokine (C-C motif) receptor 7 G protein-coupled receptor
**LCK**	206666_at	GZMK	CTL / NK-cell granzyme	pre-granzyme 3 granzyme K (serine protease granzyme 3 tryptase II) granzyme K precursor granzyme K (serine protease, granzyme 3; tryptase II)
**LCK**	206978_at	CCR2	monocyte chemokine receptor	ccr2b (ccr2) ccr2a (ccr2) ccr5 (ccr5) and ccr6 (ccr6) genes and lactoferrin (lactoferrin) gene chemokine (C-C motif) receptor 2 chemokine (C-C motif) receptor-like 2
**LCK**	207339_s_at	LTB	lymphotoxin, B-cell	DNA cosmid clones TN62 and TN82 lymphotoxin beta (TNF superfamily member 3) (LTB) transcript variant Homo sapiens DNA cosmid clones TN62 and TN82 lymphotoxin beta (TNF superfamily, member 3)
**LCK**	209670_at	TRA@	T-cell receptor	HUMTCAXB T-cell receptor active alpha-chain from JM cell line T-cell receptor active alpha-chain from JM cell lineds C-region; D-region; J-region; T-cell receptor; V-region; antigen receptor T-cell receptor alpha-chain (VDJC) T cell receptor alpha locus
**LCK**	209685_s_at	PRKCB1	protein kinase C beta	protein kinase C beta-II type (PRKCB1) d protein kinase C, beta 1
**LCK**	210031_at	CD3Z	T-cell receptor	T cell receptor zeta-chain T cell receptor zeta-chain d CD3Z antigen zeta polypeptide (TiT3 complex) T cell receptor zeta-chain T-cell receptor zeta chain precursor CD3Z antigen, zeta polypeptide (TiT3 complex)
**LCK**	210116_at	SH2D1A	T-B-cell communication	SH2D1Aformerly known as DSHP SH2 domain protein 1A Duncans disease (lymphoproliferative syndrome) SH2 domain protein 1A Duncan's disease (lymphoproliferative syndrome) SH2 domain protein 1A, Duncan's disease (lymphoproliferative
**LCK**	210915_x_at	TRBC1	T-cell receptor	T-cell specific protein T-cell receptor rearranged beta-chain V-region (V-D-J) d T cell receptor beta locus T-cell; unidentified reading frame T-cell receptor beta-chain
**LCK**	211339_s_at	ITK	T-cell specific kinase	HUMTKTCS T cell-specific tyrosine kinase IL2-inducible T-cell kinase tyrosine kinase 2024-2555 unique domain; 2556-2708 SH3 domain; 2750-3044 Sh2 domain (binds phosphotyrosine-containing proteins); 3095-3884 kiase domain (phophorylation of tyrosine residues).; putative tyrosine kinase
**LCK**	211796_s_at	TRBC1	T-cell receptor	T-cell specific protein T cell receptor beta chain (TCRBV13S1-TCRBJ2S1) d T cell receptor beta locus T-cell; unidentified reading frame T-cell receptor beta-chain
**LCK**	213193_x_at	TRBC1	T-cell receptor	T-cell specific protein T cell receptor beta locus T-cell; unidentified reading frame T-cell receptor beta-chain
**LCK**	213539_at	CD3D	T-cell receptor	ol84h02.s1 CD3D antigen delta polypeptide (TiT3 complex) CD3D antigen, delta polypeptide (TiT3 complex)
**LCK**	218805_at	GIMAP5	immune cells	th95b11.x1 hypothetical protein FLJ11296 immune associated nucleotide 4 like 1 (mouse)
**LCK**	219014_at	PLAC8	-	zl07e03.r1 hypothetical protein
**LCK**	219243_at	GIMAP4	immune cells	wg53b12.x1 hypothetical protein FLJ11110
**LCK**	64064_at	GIMAP5	immune cells	th95b11.x1 hypothetical protein FLJ11296 likely homolog of mouse immunity-associated nucleotide
**LCK**	201859_at	PRG1	-	hematopoetic proteoglycan core protein proteoglycan 1 secretory granule haematopoetic proteoglycan core protein hematopoetic proteoglycan core protein (AA 1 - 158) proteoglycan 1, secretory granule
**LCK**	202957_at	HCLS1	hematopoietic cells	HS1 geneheamatopoietic lineage cell specific protein hematopoietic cell-specific Lyn substrate 1 haematopoietic lineage cell specific protein; HS1 gene haematopoietic lineage cell protein (AA 1-486) hematopoietic cell-specific Lyn substrate 1
**LCK**	203332_s_at	INPP5D	hematopoietic cells	HSU57650 SH2-containing inositol 5-phosphatase (hSHIP) inositol polyphosphate-5-phosphatase 145kD type II 5-inositol phosphatase SH2-containing inositol 5-phosphatase inositol polyphosphate-5-phosphatase, 145kD
**LCK**	203416_at	CD53	T-cell / NK-cell	CD53 glycoprotein CD53 antigen
**LCK**	203761_at	SLA	T-cell receptor regulation	D89077 Src-like adapter protein Src-like-adapter Src-like adapter protein Src-like-adaptor
**LCK**	203879_at	PIK3CD	PI3-kinase	phosphatidylinositol 3-kinase catalytic subunit p110delta phosphatidylinositol 3-kinase catalytic subunit p110delta d phosphoinositide-3-kinase catalytic delta polypeptide PI-3 kinase catalytic subunit phosphatidylinositol 3-kinase catalytic subunit p110delta phosphoinositide-3-kinase, catalytic, delta polypeptide
**LCK**	204057_at	IRF8	interferon response	oy66c05.x1 interferon consensus sequence binding protein 1
**LCK**	204220_at	GMFG	-	za93f08.r1 glia maturation factor gamma EST glia maturation factor, gamma
**LCK**	204834_at	FGL2	-	tg73b09.x1 fibrinogen-like 2
**LCK**	204912_at	IL10RA	IL10-receptor, T-/NK-cells	U00672 interleukin-10 receptor interleukin 10 receptor alpha interleukin-10 receptor interleukin 10 receptor, alpha

Metagene Cluster	Affy_ID	Gene Symbol	Function / cell type	Description
LCK	204923_at	CXorf9	-	DNA sequence from clone 753P9 on chromosome Xq25-26.1. Contains the gene codingAminopeptidase P (EC 3.4.11.9 XAA-Pro X-Pro DNA sequence from clone 753P9 on chromosome Xq25-26.1. Contains the gene coding for Aminopeptidase P (EC 3.4.11.9 XAA-ProX-ProProlineAminoacylproline Aminopeptidase) and a novel gene. Contains ESTs STSs GSSs and a gaaa repeat polymorphi hypothetical protein likely ortholog of mouse SH3 gene SLY
LCK	205159_at	CSF2RB	subunit of GM-CSF- / IL3- / IL5- receptor	yj49e08.r1 colony stimulating factor 2 receptor beta low-affinity (granulocyte-macrophage) ESTs colony stimulating factor 2 receptor, beta, low-affinity
LCK	205269_at	LCP2	T-cell activation	76 kDa tyrosine phosphoprotein SLP-76 lymphocyte cytosolic protein 2 (SH2 domain-containing leukocyte protein of 76kD)
LCK	209083_at	CORO1A	leucocytes	actin binding protein p57 coronin-like protein (HCORO1) d coronin actin-binding protein 1A coronin, actin binding protein, 1A
LCK	209734_at	HEM1	hematopoietic cells	membrane-associated protein (HEM-1) hematopoietic protein 1 clone MGC:22 membrane-associated protein hematopoietic protein 1
LCK	209879_at	SELPLG	stimul. T-cells and myeloid cells	P-selectin glycoprotein ligand (SELPLG) gene selectin P ligand selectin P ligand
LCK	211742_s_at	EVI2B	lymphocytes	EVI2B3P gene exon and ecotropic viral integration site 2B clone MGC:145 ecotropic viral integration site 2B
LCK	212588_at	PTPRC	CD45 / leukocyte common antigen	T200 leukocyte common antigen (CD45 LC-A) protein tyrosine phosphatase receptor type C protein tyrosine phosphatase, receptor type, C
LCK	213603_s_at	RAC2	leucocytes	zd37g06.r1 HSPC022 protein EST
LCK	216250_s_at	LPXN	hematopoietic cells	leupaxin laminin alpha 3 (nicein (150kD) kalinin (165kD) BM600 (150kD) epilegrin) leupaxin similar to paxillin leupaxin
LCK	218870_at	ARHGAP15	Rho GAP	nx93e05.s1 uncharacterized bone marrow protein BM046
LCK	220330_s_at	SAMSN1	adaptor protein	wj28c05.x1 SAM domain SH3 domain and nuclear localisation signals 1 Homo sapiens chromosome 21 segment HS21C006 EST SAM domain, SH3 domain and nuclear localisation signals,
LCK	38149_at	KIAA0053	-	KIAA0053 gene for KIAA0053 gene KIAA0053 gene product
MHC-I	204806_x_at	HLA-F	MHC I component	te53h07.x1 major histocompatibility complex class I F EST major histocompatibility complex, class I, F
MHC-I	208729_x_at	HLA-B	MHC I component	wz58b02.x1 major histocompatibility complex class I B EST
MHC-I	208812_x_at	HLA-B	MHC I component	HLA class I locus C heavy chain major histocompatibility complex class I C clone MGC:110 major histocompatibility complex class I C histocompatability antigen; HLA class I heavy chain; HLA class I locus C HLA class I heavy chain major histocompatibility complex, class I, C
MHC-I	209140_x_at	HLA-B	MHC I component	wz58b02.x1 major histocompatibility complex class I B EST major histocompatibility complex, class I, B
MHC-I	210514_x_at	HLA-G	MHC I component	dJ377H14.1 (major histocompatibility complex class I G (HLA 6.0)) MHC class I antigen (HLA-G) HLA-G1 alleled HLA-G histocompatibility antigen, class I, G
MHC-I	211528_x_at	HLA-G	MHC I component	dJ377H14.1 (major histocompatibility complex class I G (HLA 6.0)) lymphocyte antigen (HLA-G2.2) d
MHC-I	211529_x_at	HLA-G	MHC I component	dJ377H14.1 (major histocompatibility complex class I G (HLA 6.0)) lymphocyte antigen (HLA-G2.1) d
MHC-I	211799_x_at	HLA-A	MHC I component	HLA class I heavy chain (HLA-Cw*1701) d
MHC-I	211911_x_at	HLA-B	MHC I component	wz58b02.x1 major histocompatibility complex class I B EST
MHC-I	213932_x_at	HLA-A	MHC I component	HLA class-I (HLA-A26) heavy chain (clone cMIY-1) major histocompatibility complex class I A
MHC-I	214459_x_at	HLA-C	MHC I component	Cw1 antigen d
MHC-I	215313_x_at	HLA-A	MHC I component	HLA class-I (HLA-A26) heavy chain (clone cMIY-1) major histocompatibility complex class I A
MHC-I	216526_x_at	HLA-C	MHC I component	HLA class I locus C heavy chain : FLJ21183 fis clone CAS11634 highly similar to HSHLACW07 for leukocyte antigen C alpha chai major histocompatibility complex class I C histocompatability antigen; HLA class I heavy chain; HLA class I locus C HLA class I heavy chain
MHC-I	217436_x_at	HLA-J	MHC I component	MHC class I HLA-J gene exons 1-8 and major histocompatibility complex class I J (pseudogene) MHC class I HLA-J antigen; cell surface antigen; cell surface glycoprotein; class I gene; inactive antigen; integral membrane protein; major histocompatibility complex heavy chain MHC class I HLA-J antigen major histocompatibility complex, class I, J (pseudogene)
MHC-I	221875_x_at	HLA-F	MHC I component	dJ377H14.9 (major histocompatibility complex class I F (CDA12)) major histocompatibility complex class I F HTG; 60S Ribosomal Protein 7A; 60S Ribosomal Protein L23A; CDA12; CpG Island; HCGIX; HLA-F; HLA-G; interferon-inducible protein 1-8U; major histocompatibility complex; MHC; MICB; P5-1; PERB11.1; RPL23A; RPL7A match: proteins: Sw:P17693 Sw:P30515 Tr:O78132 Tr:Q30716 Tr:Q30443 Tr:O62903 Tr:Q31016 Tr:Q30445 Tr:Q31012 Tr:Q31014 Tr:Q30719 Tr:Q30595 Sw:P30377 Tr:Q95483 Sw:P30517 dJ377H14.1 (major histocompatibility complex, class I, G (HLA 6.0))
STAT1	200887_s_at	STAT1	STAT signal transduction	transcription factor ISGF-3 signal transducer and activator of transcription 1 91kD transcription factor transcription factor ISGF-3 signal transducer and activator of transcription 1, 91kD
STAT1	202269_x_at	GBP1	interferon response	UI-H-BI0p-aaw-f-03-0-UI.s1 guanylate binding protein 1 interferon-inducible 67kD clone MGC:39 guanylate binding protein 1 interferon-inducible 67kD guanylate binding protein 1, interferon-inducible, 67kD
STAT1	202270_at	GBP1	interferon response	guanylate binding protein isoform I (GBP-2) guanylate binding protein 1 interferon-inducible 67kD guanylate binding protein isoform I guanylate binding protein 1, interferon-inducible, 67kD
STAT1	202307_s_at	TAP1	antigen processing	RING4 ATP-binding cassette sub-family B (MDRTAP) member 2 ATP-binding cassette sub-family B (MDR/TAP) member 2 transporter 1, ATP-binding cassette, sub-family B (MDR/TAP)
STAT1	202531_at	IRF1	STAT signal transduction	HUMIFNRF1A interferon regulatory factor 1 gene interferon regulatory factor 1
STAT1	203915_at	CXCL9	interferon response	Humig monokine induced by gamma interferon chemokine; cytokine; Humig gene; secreted protein monokine induced by gamma interferon
STAT1	204279_at	PSMB9	antigen processing	nw16h03.s1 proteasome (prosome macropain) subunit beta type 9 (large multifunctional protease 2) proteasome (prosome, macropain) subunit, beta type, 9

Metagene Cluster	Affy_ID	Gene Symbol	Function / cell type	Description
**STAT1**	204533_at	CXCL10	interferon response	HSINFGER gamma-interferon inducible early response gene (with homology to platelet proteins) small inducible cytokine subfamily B (Cys-X-Cys) member 10 small inducible cytokine subfamily B (Cys-X-Cys), member
**STAT1**	209969_s_at	STAT1	STAT signal transduction	transcription factor ISGF-3 sequence Similar to signal transducer and activator of transcription 1 91kD clone MGC:34 signal transducer and activator of transcription 1 91kD transcription factor
**STAT1**	210029_at	INDO	interferon response	interferon-gamma-inducible indoleamine 2 3-dioxygenase (IDO) interferon-gamma-inducible indoleamine 2 3-dioxygenase (IDO) d indoleamine-pyrrole 2 3 dioxygenase indole 2,3-dioxygenase indoleamine 2,3-dioxygenase (IDO) (EC 1.13.11.17) indole 2,3-dioxygenase indoleamine-pyrrole 2,3 dioxygenase
**STAT1**	210163_at	CXCL11	interferon response	interferon stimulated T-cell alpha chemoattractant precursor interferon stimulated T-cell alphachemoattractant precursor small inducible cytokine subfamily B (Cys-X-Cys) member 11 chemokine; I-TAC interferon stimulated T-cell alpha chemoattractant precursor
**STAT1**	211122_s_at	CXCL11	interferon response	interferon stimulated T-cell alpha chemoattractant precursor small inducible cytokine subfamily B (Cys-X-Cys) member 11 chemokine; I-TAC interferon stimulated T-cell alpha chemoattractant precursor small inducible cytokine subfamily B (Cys-X-Cys), member
**STAT1**	219209_at	IFIH1	interferon response	zo23f04.s1 melanoma differentiation associated protein-5 accessory proteins BAP31/BAP29 EST
**STAT1**	AFFX-HUMISGF3A/M97935_3_at	- - -	interferon response	M97935 Homo sapiens transcription factor ISGF-3 mRNA, complete cds (_5, _MA, MB, _3 represent transcript regions 5 prime, MiddleA, MiddleB, and 3 prime respectively)
**Interferon**	202086_at	MX1	interferon inducible	p78 protein myxovirus (influenza) resistance 1 homolog of murine (interferon-inducible protein p78) p78 protein p78 protein myxovirus (influenza) resistance 1, homolog of murine (interferon-inducible protein p78) myxovirus (influenza virus) resistance 1, interferon-inducible
**Interferon**	202411_at	IFI27	interferon inducible	HSP27 p27 interferon alpha-inducible protein 27 interferon, alpha-inducible protein 27
**Interferon**	202869_at	OAS1	interferon inducible	(2-5'') oligo A synthetase E gene 2 5-oligoadenylate synthetase 1 (40-46 kD) (OAS1) transcript variant E 2',5'-oligoadenylate synthetase 1 (40-46 kD)
**Interferon**	203153_at	IFIT1	interferon inducible	HUMII56KD interferon-inducible 56 Kd protein interferon-induced protein with tetratricopeptide repeats 1
**Interferon**	204415_at	G1P3	interferon inducible	HSU22970 interferon-inducible peptide (6-16) gene interferon alpha-inducible protein (clone IFI-6-16) (G1P3) transcript variant interferon alpha-inducible protein (clone IFI-6-16) gene 6-16; interferon; interferon-inducible; interferon stimulatable response element (ISRE); minisatellite; expressed minisatellite; alternative splicing alternatively spliced using the second alternative exon 2 interferon-inducible peptide precursor interferon, alpha-inducible protein (clone IFI-6-16)
**Interferon**	204439_at	IFI44L	interferon inducible	expressed in osteoblast hypothetical protein expressed in osteoblast GS3686 The submitters designated this product as GS3686 hypothetical protein, expressed in osteoblast
**Interferon**	204747_at	IFIT3	interferon inducible	CIG49 (cig49) interferon-induced protein with tetratricopeptide repeats 4
**Interferon**	204972_at	OAS2	interferon inducible	71 kDa 25'' oligoadenylate synthetase (p69 2-5A synthetase) 2-5oligoadenylate synthetase 2 (OAS2) transcript variant 2'-5'oligoadenylate synthetase 2 2'-5'-oligoadenylate synthetase 2 (69-71 kD)
**Interferon**	205483_s_at	G1P2	interferon inducible	zx57e04.r1 interferon-stimulated protein 15 kDa interferon-stimulated protein, 15 kDa
**Interferon**	205552_s_at	OAS1	interferon inducible	1.6Kb 2-5A synthetase induced by interferon 2 5-oligoadenylate synthetase 1 (40-46 kD) (OAS1) transcript variant E 2' 5'-oligoadenylate synthetase 1 2',5'-oligoadenylate synthetase 1 (40-46 kD)
**Interferon**	213797_at	RSAD2	interferon inducible	cig5 sequence cig5 partial sequence
**Interferon**	214453_s_at	IFI44	interferon inducible	genehepatitis C-associated microtubular aggregate protein p44 interferon-induced hepatitis C-associated microtubular aggregate protein (44kD) hepatitis C-associated microtubular aggregate protein; p44 interferon-induced, hepatitis C-associated microtubular aggregate protein (44kD)
**Interferon**	218400_at	OAS3	interferon inducible	2-5oligoadenylate synthetase 3 2'-5'-oligoadenylate synthetase 3 (100 kD)
**Interferon**	218986_s_at	FLJ20035	-	DKFZp434E1722_s1 hypothetical protein FLJ20035 EST
