# Supplementary material for: T-cell metagene predicts a favorable prognosis in estrogen receptor-negative and HER2-positive breast cancers
Source: Breast Cancer Res. 2009 Mar 9;11(2):R15. doi: 10.1186/bcr2234 (PMC2688939; doi:10.1186/bcr2234)
Supplement: Additional file 6 — An Adobe file containing two figures presenting the relationship of the expression of the immune-system-related metagenes with estrogen receptor status, human epidermal growth factor receptor 2 status and the presence of stem-cell like markers in all 1,781 samples from all datasets. Unsupervised clustering analysis of the samples using the immune-related metagenes is presented in Supplemental figure S2a. Scatter plots of the five metagenes representing the major clusters are given in Supplemental figure S2b. [file bcr2234-S6.pdf]

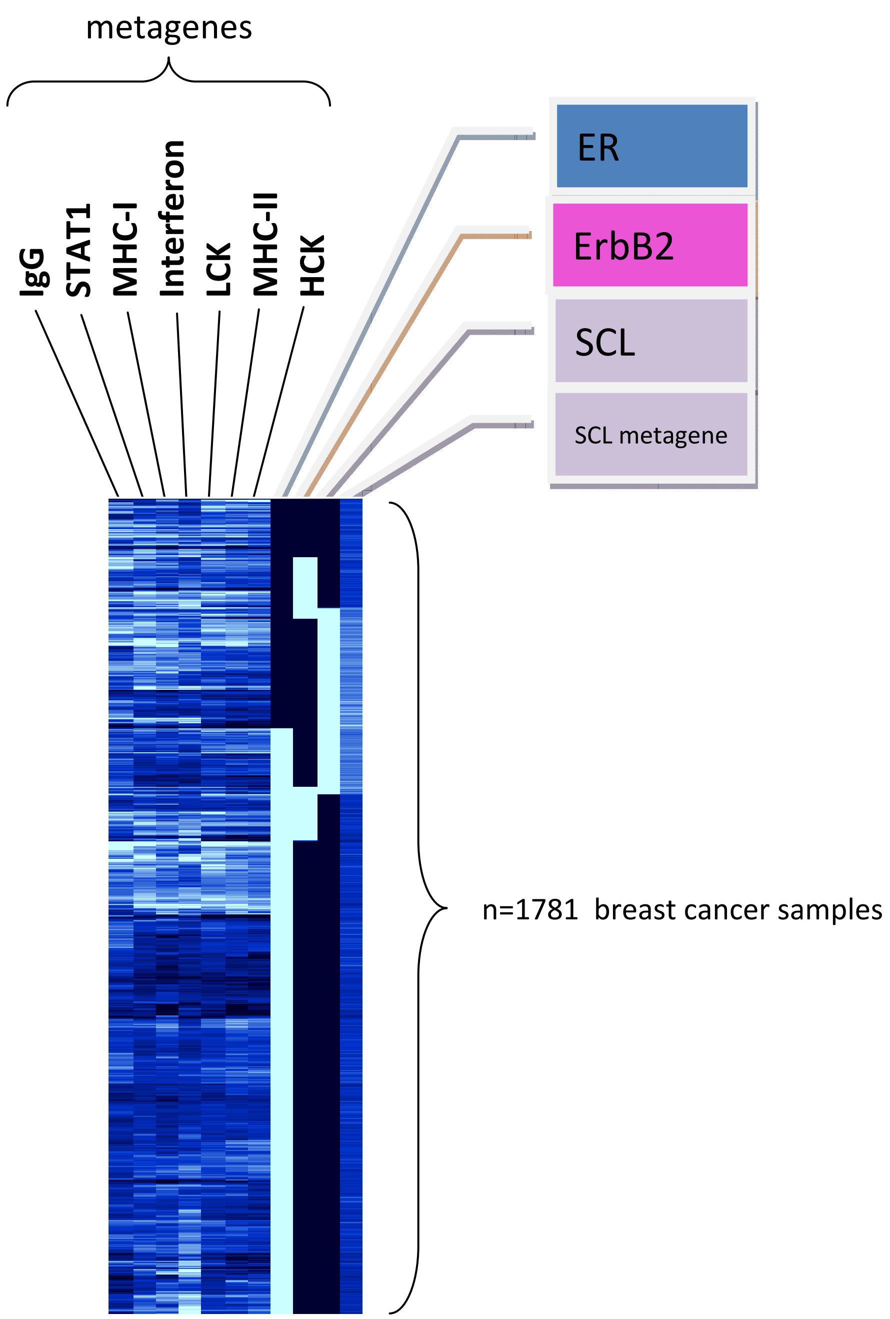

Suppl. Figure S2A: Relationship of the expression of the immune system related "metagenes" with ER status, Her2 status and the presence of "stem cell like" (SCL) markers in 1781 samples from all datasets.

A) Unsupervised clustering analysis of the samples using the immune related "metagenes" suggests that considerable amounts of immune cells seem to be present among all different subtypes of tumors.

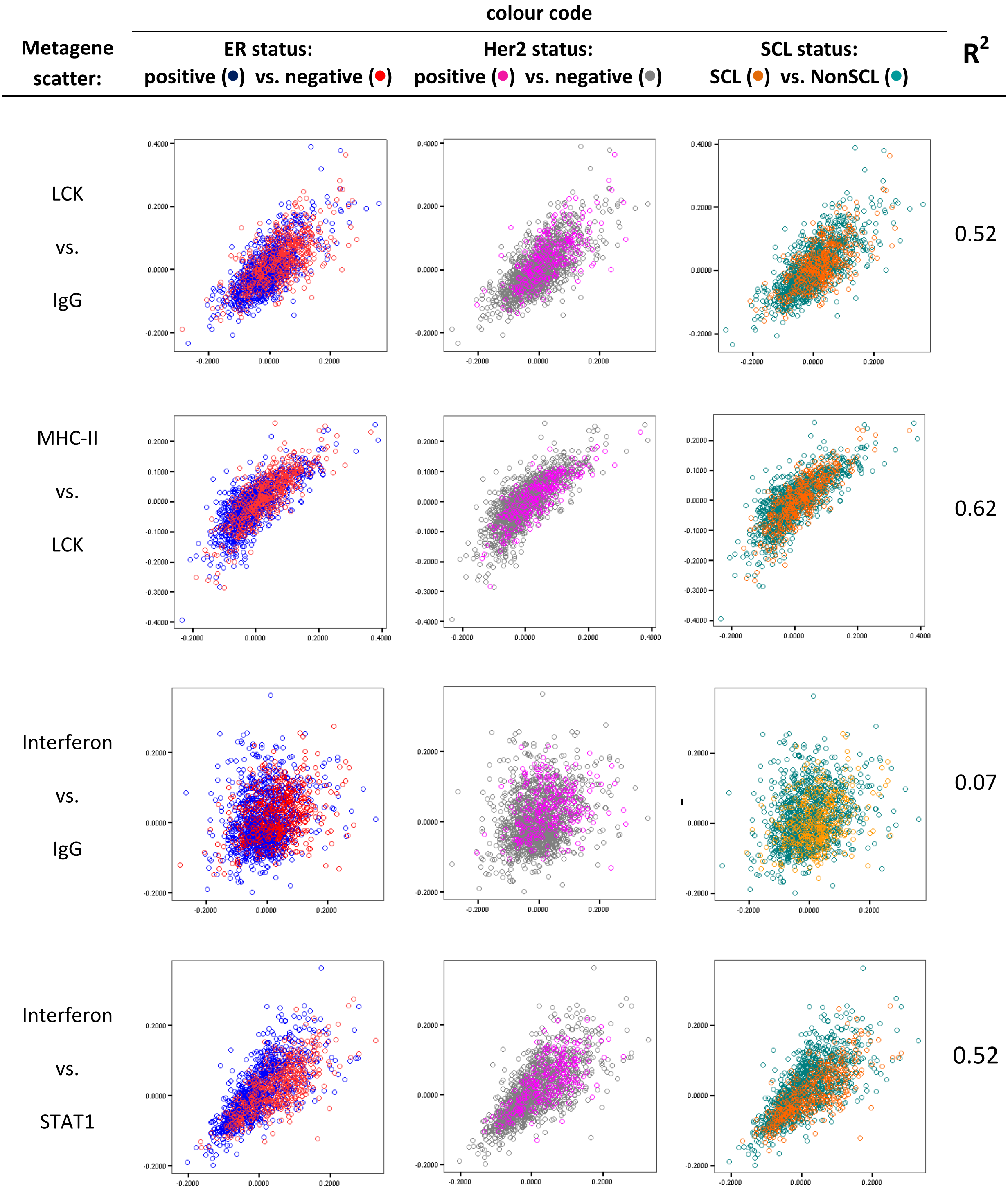

Suppl. Figure S2B: Relationship of the expression of the immune system related "metagenes" with ER status, Her2 status and the presence of "stem cell like" (SCL) markers in 1781 samples from all datasets.

Scatter plots comparing five metagenes representing the major clusters ("LCK", "IgG","MHC-II", "Interferon", "STAT1").

Relationship of the expression of the immune system related "metagenes" with ER status, HER2 status and the presence of "stem cell like" (SCL) markers in all 1781 samples from all datasets:

A) Unsupervised clustering analysis of the samples using the immune related “metagenes” suggests that considerable amounts of immune cells seem to be present among all different subtypes of tumors.

B) Scatter plots comparing five metagenes representing the major clusters ("LCK", "IgG","MHC-II", "Interferon", "STAT1"). ER status and HER2 status of the tumor samples and positivity for "stem cell like" (SCL) markers are given by different colors in the three columns of the respective scatter plots. The scatter of LCK and IgG (as well as MHC-II) metagenes showed a correlation (R^2: 0.52 and 0.62, respectively) which was not observed between the Interferon and IgG metagenes (R^2=0.07). This could suggest a parallel infiltration by both T and B cells into those tumors which are characterized by high expression of both metagenes. On the other hand the Interferon and STAT1 metagenes are also correlated (R^2=0.52) which might represent an interferon response of tumor cells or other cell types in the respective samples. In general no perfect relationship with the subtype of tumors is seen in these scatter plots. However, the plots in the first column suggest that ER negative tumors (red dots) display a somewhat higher expression of the IgG and STAT1 metagenes.
