# Supplementary material for: T-cell metagene predicts a favorable prognosis in estrogen receptor-negative and HER2-positive breast cancers
Source: Breast Cancer Res. 2009 Mar 9;11(2):R15. doi: 10.1186/bcr2234 (PMC2688939; doi:10.1186/bcr2234)
Supplement: Additional file 7 — An Adobe file containing a table that presents the results from multivariate Cox regression of immune-system-related metagenes in relation to disease-free survival of breast cancer patients (n = 1,263). [file bcr2234-S7.pdf]

Multivariate Cox regression of immune system related metagenes in relation to disease free survival of breast cancer patients (n=1263)

Metagene		P Value*	HR	(95% CI)
LCK	high vs low[§]	**0.013**	0.589	(0.389 - 0.893)
HCK	high vs low	0.447	1.150	(0.802 - 1.651)
IgG	high vs low	0.687	1.060	(0.799 - 1.405)
MHC-II	high vs low	0.998	1.001	(0.620 - 1.615)
MHC-I	high vs low	0.106	0.771	(0.562 - 1.057)
STAT1	high vs low	0.131	1.304	(0.924 1.841)
Interferon	high vs low	0.543	1.085	(0.834 - 1.411)

* significant values are given in bold type

[§] stratified by the highest quartile compared to the rest of the samples
