# Supplementary material for: T-cell metagene predicts a favorable prognosis in estrogen receptor-negative and HER2-positive breast cancers
Source: Breast Cancer Res. 2009 Mar 9;11(2):R15. doi: 10.1186/bcr2234 (PMC2688939; doi:10.1186/bcr2234)
Supplement: Additional file 8 — An Adobe file containing a figure that presents a Kaplan–Meier plot that presents the prognostic value of the IgG metagene in breast cancer patients. [file bcr2234-S8.pdf]

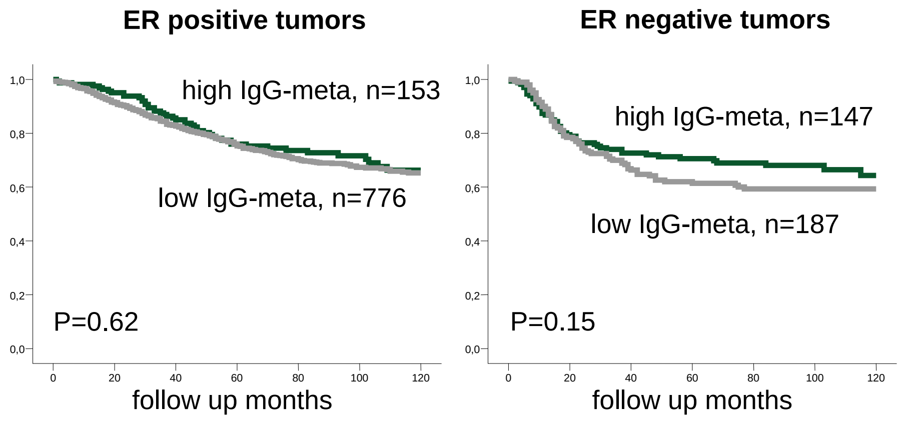

Prognostic value of the IgG metagene in breast cancer patients.

The samples of the combined dataset were stratified according to the highest quartile of expression of the IgG metagene. Kaplan Meier analyses of disease free survival were performed separately in ER positive and ER negative subgroups of tumors (n=1263).
