# Supplementary material for: T-cell metagene predicts a favorable prognosis in estrogen receptor-negative and HER2-positive breast cancers
Source: Breast Cancer Res. 2009 Mar 9;11(2):R15. doi: 10.1186/bcr2234 (PMC2688939; doi:10.1186/bcr2234)
Supplement: Additional file 9 — An Adobe file containing a figure that presents the relationship of the expression of immune-system-related metagenes and response to neoadjuvant chemotherapy. Pretherapeutic breast cancer samples (n = 198) from patients treated with neoadjuvant chemotherapy were stratified according to the estrogen receptor status of the tumor and were analyzed for expression of IgG and lymphocyte-specific kinase metagenes in a scatter plot. [file bcr2234-S9.pdf]

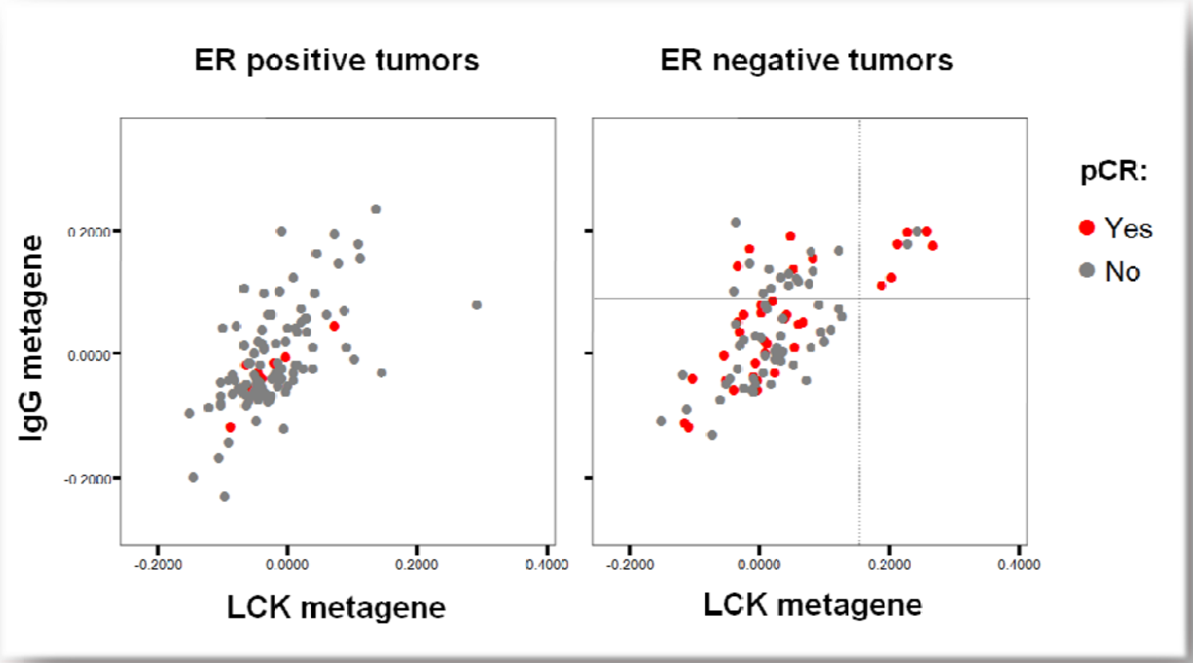

Relationship of the expression of immune system related metagenes and response to neoadjuvant chemotherapy.

Pre-therapeutic breast cancer samples (n=198) from patients treated with neadjuvant chemotherapy were stratified according to the ER status of the tumor and analyzed for expression of IgG and LCK metagenes in a scatter plot. Patients which achieved a pathological complete response (pCR) are represented by red dots. 6 of 8 samples (75%) with highest expression of both metagenes in the ER negative group displayed a pCR as compared to 37 cases with pCR among all 92 ER negative samples (40.2%, P=0.057). When the confounding effect of the ER status was not taken into account and ER positive samples were pooled with ER negative tumors only 45 of all 198 samples (22.7%) achieved a pCR (P=0.002).
