# Supplementary material for: T-cell metagene predicts a favorable prognosis in estrogen receptor-negative and HER2-positive breast cancers
Source: Breast Cancer Res. 2009 Mar 9;11(2):R15. doi: 10.1186/bcr2234 (PMC2688939; doi:10.1186/bcr2234)
Supplement: Additional file 10 — An Adobe file containing a table that presents a list of the tissue and cell line samples from Figure 2. Gene Expression Omnibus database accession numbers of expression data from Su and colleagues [GEO:GSE1133] [31] and a description of the samples from immunological cell types and tissues as ordered in Figure 2 (left to right) are given. [file bcr2234-S10.pdf]

Tissue and cell line samples from Figure 2.

GEO accession numbers of expression data of Su et al. (GSE1133) and description of the samples from immunological cell types and tissues as ordered in Figure 2 (left to right) are given.

GEO	Sample
GSM18905	fetal liver
GSM18906	fetal liver
GSM18897	"leukemia, chronic Myelogenous K-562"
GSM18898	"leukemia, chronic Myelogenous K-562"
GSM18867	WHOLEBLOOD
GSM18868	WHOLEBLOOD
GSM18869	bone marrow-CD33Myeloid
GSM18870	bone marrow-CD33Myeloid
GSM18871	peripheral blood-CD14Monocytes
GSM18872	peripheral blood-CD14Monocytes
GSM18885	bone marrow-CD34
GSM18886	bone marrow-CD34
GSM18889	721_BLymphoblasts
GSM18890	721_BLymphoblasts
GSM18875	peripheral blood-CD56NKCells
GSM18876	peripheral blood-CD56NKCells
GSM18878	peripheral blood-CD4TCells
GSM18877	peripheral blood-CD4TCells
GSM18879	peripheral blood-CD8TCells
GSM18880	peripheral blood-CD8TCells
GSM18887	leukemia lymphoblastic (MOLT-4)
GSM18888	leukemia lymphoblastic (MOLT-4)
GSM18891	LymphomaRaji
GSM18892	LymphomaRaji
GSM18893	"leukemia, promyelocytic-HL-60"
GSM18894	"leukemia, promyelocytic-HL-60"
GSM18895	lymphomaburkittsDaudi
GSM18896	lymphomaburkittsDaudi
GSM18883	bone marrow-CD105Endothelial
GSM18884	bone marrow-CD105Endothelial
GSM18907	bone marrow-CD71EarlyErythroid
GSM18908	bone marrow-CD71EarlyErythroid
GSM18873	peripheral blood-BDCA4DentriticCells
GSM18874	peripheral blood-BDCA4DentriticCells
GSM18881	peripheral blood-CD19BCells
GSM18882	peripheral blood-CD19BCells
GSM18899	thymus
GSM18900	thymus
GSM18901	Tonsil
GSM18902	Tonsil
GSM18903	lymph node
GSM18904	lymph node
GSM18909	bone marrow
GSM18910	bonemarrow
